# Synthesis and crystal structures of two solvates of 1-{[2,6-bis­(hy­droxy­meth­yl)-4-methyl­phen­oxy]meth­yl}-3,5-bis­{[(4,6-di­methyl­pyridin-2-yl)amino]meth­yl}-2,4,6-tri­ethyl­benzene

**DOI:** 10.1107/S2056989023009155

**Published:** 2023-10-24

**Authors:** Manuel Stapf, Ute Schmidt, Wilhelm Seichter, Monika Mazik

**Affiliations:** a Institut für Organische Chemie, Technische Universität Bergakademie Freiberg, Leipziger Str. 29, 09599 Freiberg, Germany; Universidade de Sâo Paulo, Brazil

**Keywords:** crystal structures, tripodal mol­ecule, hydrogen bonding, C—H⋯π and π–π inter­actions

## Abstract

In the crystal structures of the formamide monosolvate (**1a**) and the *n*-propanol/H_2_O solvate/hydrate (**1b**) of the title compound, C_38_H_50_N_4_O_3_ (**1**), the tripodal host mol­ecule adopts a conformation in which the substituents attached to the central benzene ring are arranged in an alternating order above and below the ring plane. As a result of the different nature of the involved guest species, the crystal components in **1a** create a three-dimensional supra­molecular architecture, while the crystal structure of **1b** consists of two-dimensional supra­molecular aggregates extending parallel to the crystallographic *ab* plane.

## Abstract 2

In the crystal structures of the formamide monosolvate (**1a**) and the *n*-propanol/water solvate/hydrate (**1b**), the host mol­ecules adopt similar geometries with an alternating arrangement of the substituents above and below the plane of the central arene ring.

## 1. Chemical context

1,3,5-Trisubstituted 2,4,6-tri­ethyl­benzene derivatives with functionalized side-arms can serve as artificial receptors for mol­ecular recognition of carbohydrates. In addition to the development of acyclic receptor mol­ecules, the tri­ethyl­benzene scaffold was found to be valuable for the construction of macrocyclic systems. The possibilities for functionalization of acyclic and macrocyclic mol­ecules of this type are manifold, allowing the synthesis of a whole range of compounds for systematic binding studies. Some examples of suitable functional groups, which can act as recognition units and have been considered in our studies, are heteroaromatic units such as pyridine-, pyrimidine- (Lippe *et al.*, 2015 ▸), pyrazole- (Koch *et al.*, 2016 ▸), purine- (Kaiser *et al.*, 2019 ▸) or phenanthroline-based recognition groups (Köhler *et al.*, 2020 ▸), (cyclo)­alkyl­amino groups (Stapf *et al.*, 2020*a* ▸; Leibiger *et al.*, 2022 ▸) as well as subunits containing hy­droxy groups. Among the mol­ecules with the latter groups, studies of the binding properties of acyclic (Mazik & Kuschel, 2008*a* ▸) and macrocyclic (Amrhein *et al.*, 2016 ▸) compounds bearing a hy­droxy­methyl group at the tri­ethyl­benzene core should be mentioned. These binding studies included NMR spectroscopic titrations and microcalorimetric investigations (ITC experiments). Similarly, chip calorimetry experiments were performed with one of our receptor compounds possessing [1-(hy­droxy­meth­yl)cyclo­pent-1-yl]amino moieties (Lerchner *et al.*, 2022 ▸). Further compounds bearing hy­droxy groups, whose crystal structures we have recently discussed (Stapf *et al.*, 2020*b* ▸, 2022 ▸), are currently being investigated for their ability to act as receptors for carbohydrates. In this article, we describe the crystal structures of the formamide monosolvate and the *n*-propanol/H_2_O solvate/hydrate of compound **1** containing the 2,6-bis­(hy­droxy­meth­yl)-4-methyl­phen­oxy moiety, which represents a new structural unit for the design of carbohydrate receptors. 
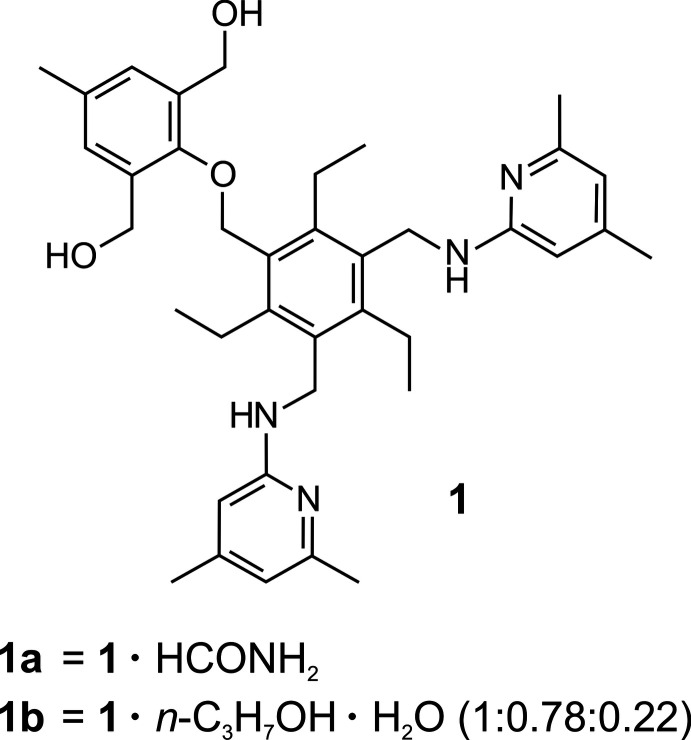


## 2. Structural commentary

**Figure 1:**
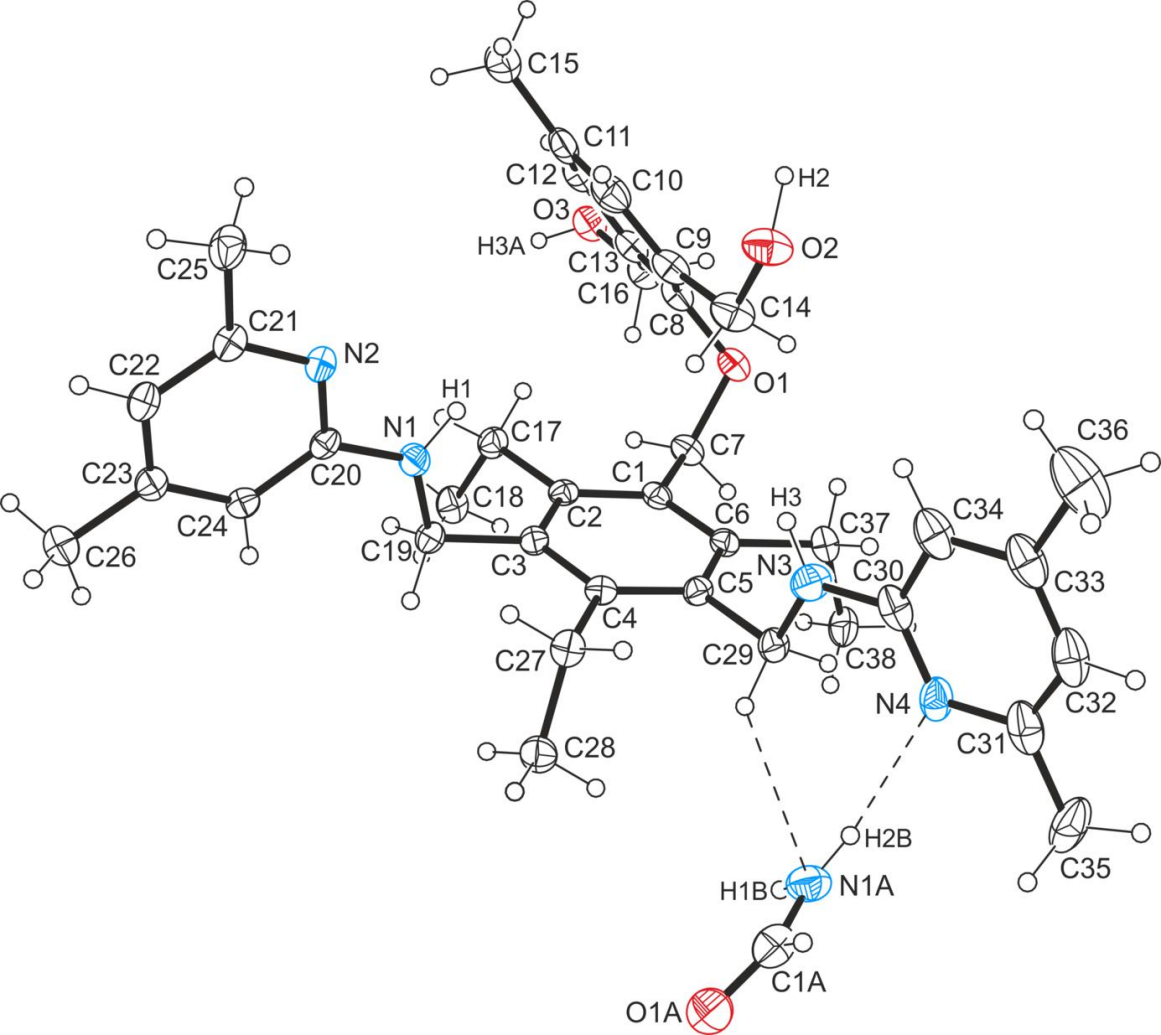
Perspective view of the host–guest complex **1a** including atom labelling. Anisotropic displacement ellipsoids are drawn at the 50% probability level. Inter­molecular hydrogen bonds between the host mol­ecule and the formamide are shown as dashed lines.

**Figure 2:**
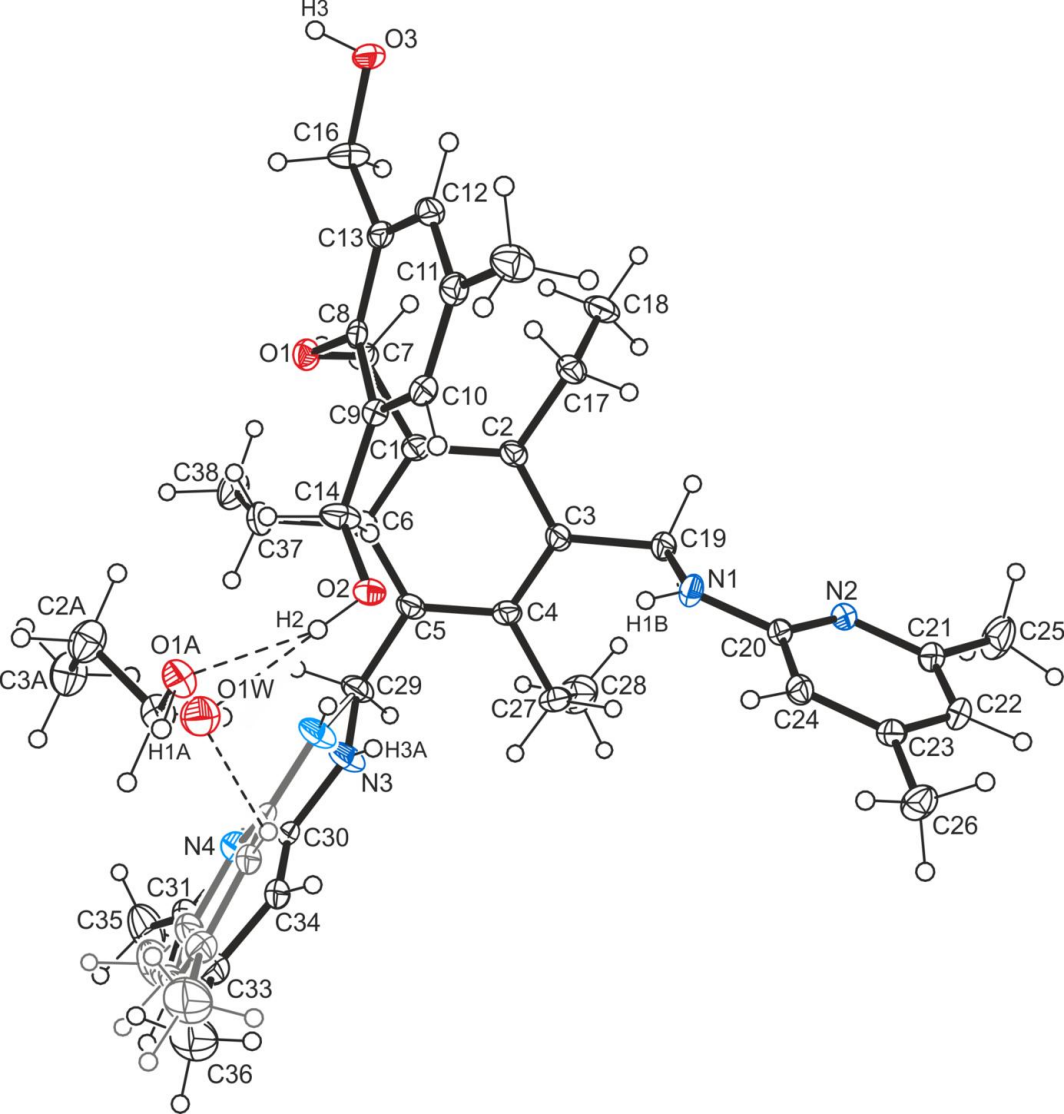
Perspective view of the host–guest complex **1b** including atom labelling. Anisotropic displacement ellipsoids are drawn at the 50% probability level. Inter­molecular hydrogen bonds are shown as dashed lines.

The formamide solvate (**1a**) and the *n*-propanol/H_2_O solvate/hydrate (**1b**) of the title compound, C_38_H_50_N_4_O_3_, crystallize in the triclinic system (*P* 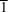 , *Z* = 2). The model for the least-squares refinement includes positional disorder for one of the (4,6-di­methyl­pyridin-2-yl)amino moieties of the structure of **1b** with occupancies of 0.78/0.22. The perspective views of the host–guest complexes shown in Fig. 1 ▸ and Fig. 2 ▸ reveal similar geometries of the tripodal host mol­ecule with the three functionalized side-arms located on one side of the central benzene ring, while the ethyl substituents are oriented in the opposite direction. The inclination angles of the aromatic rings of the substituents with reference to the central benzene ring are 50.9 (1), 85.5 (1), 87.2 (1)° for **1a** and 61.3 (1), 81.3 (1), 80.7 (1)/88.4 (3)° for **1b**. Despite the large number of strong donor/acceptor sites in the host mol­ecule, only three intra­molecular C—H⋯O hydrogen bonds [*d*(H⋯O) = 2.42–2.49 Å; Table 1 ▸] are observed in the crystal of **1a**. Consequently, the irregular but compact geometry of the mol­ecule is likely to be caused by inter­molecular inter­actions and packing effects. The conformation of the receptor mol­ecule in the crystal of **1b** is stabilized by four relatively short intra­molecular C—H⋯O and C—H⋯N hydrogen bonds [*d*(H⋯O) = 2.41–2.49 Å, *d*(H⋯N) = 2.55 Å; Table 2 ▸].

## 3. Supra­molecular features

**Figure 3:**
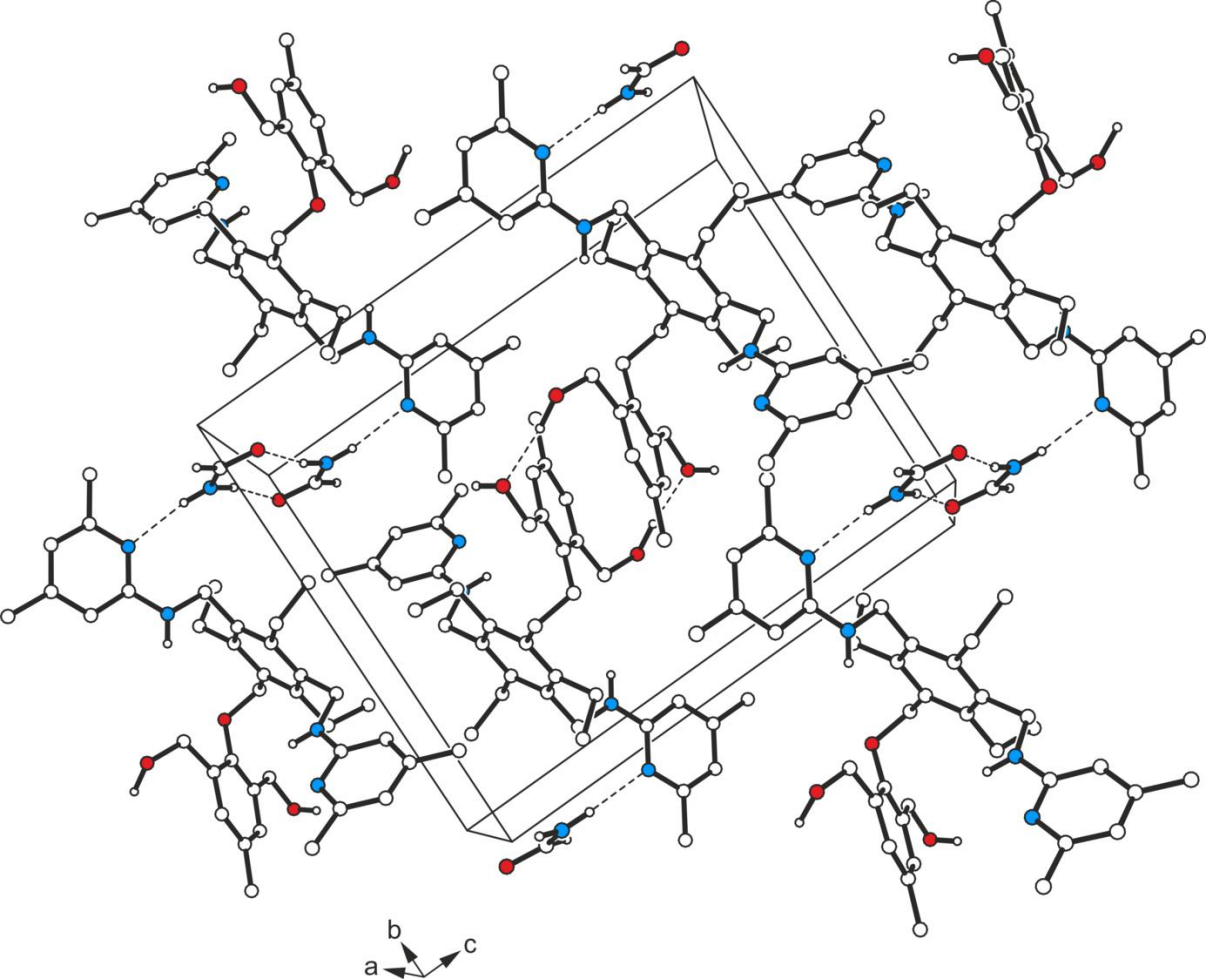
Packing diagram of the formamide monosolvate of the title compound **1a**. Dashed lines represent hydrogen-bond inter­actions.

In the complex structure of **1a** (Fig. 3 ▸), the formamide mol­ecule is connected to the host mol­ecule by an N—H⋯N hydrogen bond [*d*(H⋯N) = 2.12 (1) Å] and a weak C—H⋯N bond (Desiraju & Steiner, 1999 ▸) [*d*(H⋯N) = 2.59 Å]. With the exception of the amino hydrogen H3, which for sterical reasons seems to be excluded from non-covalent bonding, all other strong donors participate in mol­ecular association comprising O—H⋯O [*d*(H⋯O) = 2.26 Å], O—H⋯N [*d*(H⋯N) = 2.00 (1) Å] and N—H⋯O type [*d*(H⋯O) = 2.46 (2) Å] hydrogen bonds. The pattern of inter­molecular bonding is completed by C—H⋯O inter­actions [*d*(H⋯O) = 2.52–2.63 Å], C—H⋯π contacts (Nishio *et al.*, 2009 ▸, 2012 ▸) [*d*(H⋯*Cg*) = 2.71–2.81 Å] and π–π stacking (Dance, 2004 ▸; Salonen *et al.*, 2011 ▸) [*Cg*⋯*Cg* distance = 3.475 (1) Å], the latter formed by the hy­droxy­methyl-substituted aromatic rings of inversion-related mol­ecules. Within this three-dimensional supra­molecular network, the solvent mol­ecules form N—H⋯O bonded dimers [*d*(H⋯O) = 2.01 (1) Å] of the graph-set motif 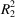 (8) (Etter, 1991 ▸; Bernstein *et al.*, 1995 ▸).

**Figure 4:**
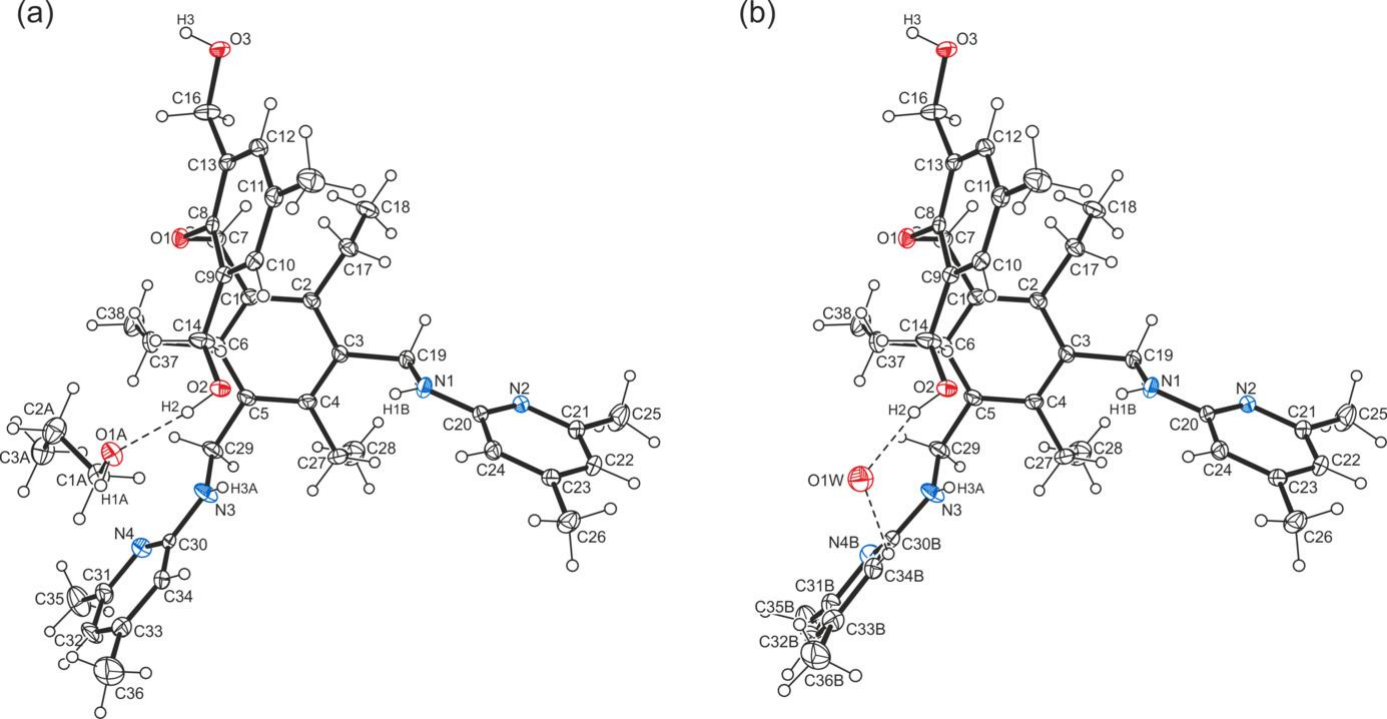
Perspective views of the structures of the *n*-propanol solvate (*a*) and the monohydrate (*b*) of the title compound.

**Figure 5:**
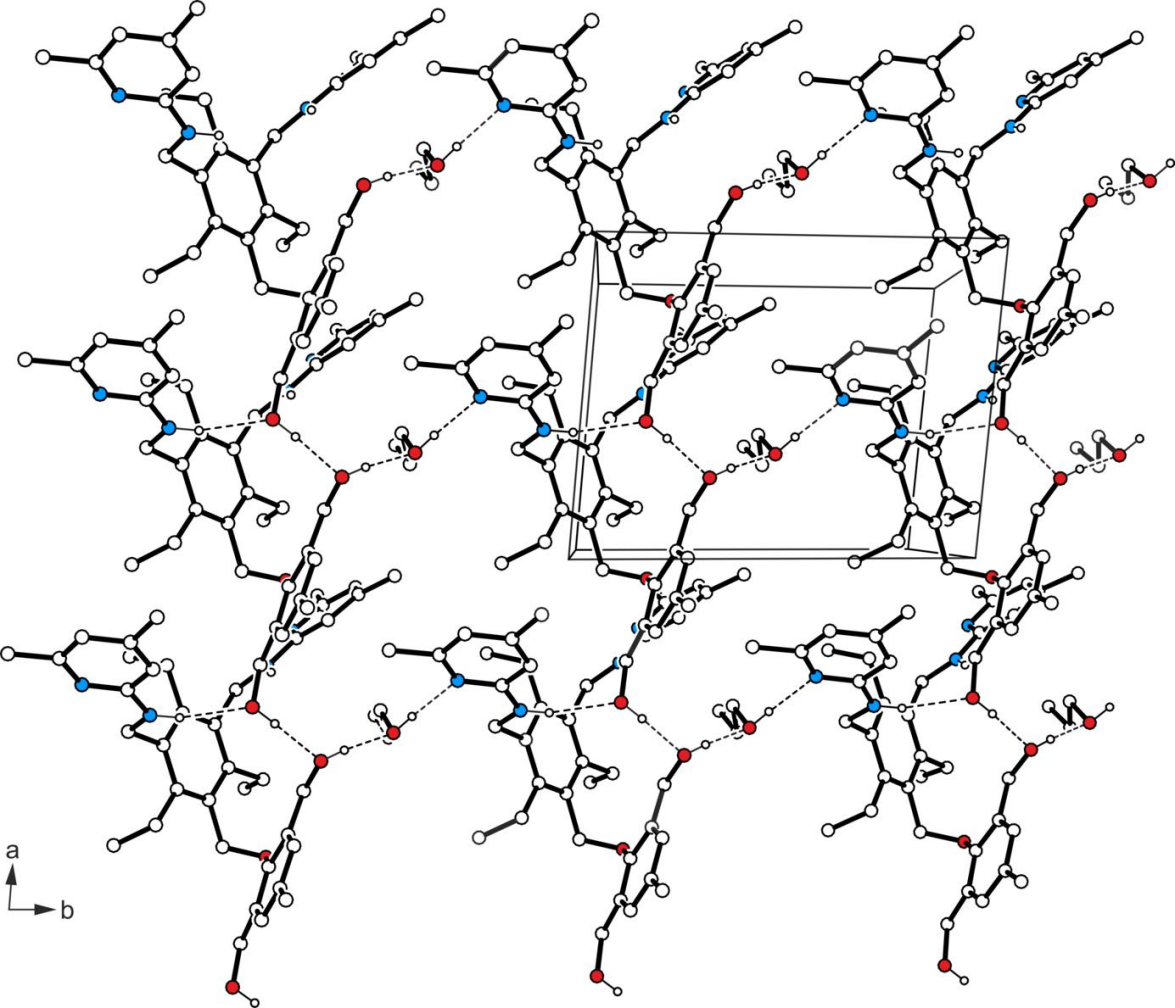
Packing diagram of the host–guest complex **1b** looking in the crystallographic *c*-axis direction. For clarity, the water mol­ecules are not shown. Dashed lines represent hydrogen-bond inter­actions.

The colourless rod-like crystals obtained from *n*-propanol proved to be an inclusion compound of **1** with *n*-PrOH and H_2_O possessing a host/guest stoichiometric ratio of 1:0.78:0.22. The model for least-squares refinement assumes partial occupancies for the alcohol and water mol­ecules, *i.e.* the solvent species are distributed in a statistical manner in the voids of the host lattice. Despite the presence of strong donors/acceptors, the disordered moiety of the host hardly participates in mol­ecular association. Only the minor disorder component of this residue is involved in any inter­molecular inter­actions, by forming a weak C—H⋯O bond to the water oxygen [*d*(H⋯O) = 2.16 Å] (see Fig. 4 ▸ *b*). As shown in Fig. 4 ▸ *a*, the oxygen atom of the alcohol mol­ecule is linked to one of the hy­droxy hydrogens of the host [O2—H2⋯O1*A*, *d*(H⋯O) = 1.86 (2) Å]. In an analogous way, this hydrogen acts as a donor site for hydrogen bonding to the water mol­ecule [O2—H2⋯O1*W*, *d*(H⋯O) = 1.97 (2) Å]. Unfortunately, the positions of the water hydrogen atoms could not be obtained from the difference electron-density map, so that the complete pattern of hydrogen bonding in the crystal of **1b** could not be elucidated. Nevertheless, a striking motif of hydrogen bonds is present, involving a total of three hy­droxy groups of the host and the propanol mol­ecules [oxygen atoms O1*A*, O2 and O3; *d*(H⋯O/N) = 1.86 (2)–2.21 (1) Å]. They form chain-like synthons in the direction of the *b* axis, bounded by an amine H and a ring N atom, and can be described by the graph set 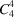 (10) (Fig. 5 ▸). Taking into account these inter­actions, the crystal structure (Fig. 5 ▸) can be regarded as being composed of layered supra­molecular aggregates extending parallel to the crystallographic *ab* plane. As the surfaces of the two-dimensional aggregates are defined by the non-polar mol­ecular parts, inter­layer inter­actions are restricted to van der Waals forces.

## 4. Database survey

The search in the Cambridge Structural Database (CSD, Version 5.44, update April 2023; Groom *et al.*, 2016 ▸) for 2,4,6-tri­ethyl­benzene-based tripodal mol­ecules containing two (4,6-di­methyl­pyridin-2-yl)amino­methyl moieties resulted in several hits, which are described below. Particularly noteworthy is 1,3,5-tris­[(4,6-di­methyl­pyridin-2-yl)amino­meth­yl]-2,4,6-tri­ethyl­benzene, which has proven to be an effective receptor mol­ecule for complex formation with methyl *β*-d-gluco­pyran­oside in the solid state (LAJZOP; Köhler *et al.*, 2020 ▸). In the ethanol solvate of this host compound (RAJZAE; Mazik *et al.*, 2004 ▸), its (4,6-di­methyl­pyridin-2-yl)amino units are arranged in a ‘two *up*/one *down*’ pattern with respect to the benzene plane. The heterocyclic units of the 1-[*N*-(1,10-phenanthrolin-2-ylcarbon­yl)amino­meth­yl]-3,5-bis­[(4,6-di­methyl­pyridin-2-yl)amino­meth­yl]-2,4,6-tri­ethyl­benz­ene diethyl ether solvate trihydrate (ROKJEH, ROKJEH01; Mazik & Hartmann, 2008 ▸; Mazik *et al.*, 2009 ▸) form a binding pocket in which the three water mol­ecules are located. This aggregate is stabilized by a total of eight hydrogen bonds. The crystal structures of the monohydrate and the methanol solvate of {1-[(3,5-bis­[(4,6-di­methyl­pyridin-2-yl)amino­meth­yl]-2,4,6-tri­ethyl­benz­yl)amino]­cyclo­pent­yl}methanol (CAD­TAG, CADTEK; Stapf *et al.*, 2020*b* ▸) are composed of structurally similar dimers of 1:1 host–guest complexes. In the crystal structure of the diethyl ether solvate of 1-(bromo­meth­yl)-3,5-bis­[(4,6-di­methyl­pyridin-2-yl)amino­meth­yl]-2,4,6-tri­ethyl­benzene (BIYTOT; Mazik & Kuschel, 2008*b* ▸), the host mol­ecule adopts a conformation with a complete *up*–*down* alternation of the side chains on the benzene ring (for discussions on conformations of 1,3,5-tri­substituted 2,4,6-tri­alkyl­benzene-based compounds, see: Koch *et al.*, 2017 ▸; Schulze *et al.*, 2017 ▸).

## 5. Synthesis and crystallization

A suspension of 2,6-bis­(hy­droxy­meth­yl)-4-methyl­phenol (102 mg, 0.61 mmol) and potassium carbonate (142 mg, 1.03 mmol) in 30 mL of THF/CH_3_CN (1:1, *v*/*v*) was stirred for 30 minutes. Subsequently, a solution of 1-(bromo­meth­yl)-3,5-bis­[(4,6-di­methyl­pyridin-2-yl)amino­meth­yl]-2,4,6-tri­ethyl­ben­z­ene (265 mg, 0.51 mmol) in 30 mL of THF/CH_3_CN (1:1, *v*/*v*) was added dropwise and the resulting mixture was stirred at room temperature and under the exclusion of light (the progress of the reaction was monitored by TLC). After filtration, the solvents were evaporated at reduced pressure and the yellow oil was treated with THF/water. The oil was separated from the aqueous phase and dissolved again in THF, dried over MgSO_4_ and the solvent was removed. By treating the oily residue with diethyl ether/*n*-hexane, the product was obtained as a white solid in 88% yield (271 mg, 0.44 mmol). Crystals of the title compound suitable for single crystal X-ray diffraction were grown by slow evaporation of an ethyl acetate/formamide (1:1, *v*/*v*) solution (**1a**) or a *n*-propanol solution (**1b**) at ambient temperature.

*Analysis data*: m.p. = 472 K; ^1^H NMR (600 MHz, CDCl_3_, ppm): *δ* = 1.12 (*t*, 6H, ^3^ *J* = 7.5 Hz, CH_2_C**H**_3_), 1.23 (*t*, 3H, ^3^ *J* = 7.5 Hz, CH_2_C**H**_3_), 2.23 (*s*, 6H, ArC**H**_3_), 2.29 (*s*, 3H, ArC**H**_3_), 2.34 (*s*, 6H, ArC**H**_3_), 2.69 (*q*, 4H, ^3^ *J* = 7.5 Hz, C**H**_2_CH_3_), 2.74 (*q*, 2H, ^3^ *J* = 7.5 Hz, C**H**_2_CH_3_), 4.33 (*br*, 4H, C**H**_2_NH), 4.46 (*s*, 4H, C**H**_2_OH), 5.20 (*s*, 2H, C**H**_2_OAr), 6.10 (*s*, 2H, Ar**H**), 6.34 (*s*, 2H, Ar**H**), 7.11 (*s*, 2H, Ar**H**); ^13^C NMR (151 MHz, CDCl_3_, ppm): *δ* = 16.6 (CH_2_ **C**H_3_), 16.8 (CH_2_ **C**H_3_), 20.8 (Ar**C**H_3_), 21.1 (Ar**C**H_3_), 22.6 (**C**H_2_CH_3_), 23.0 (**C**H_2_CH_3_), 24.0 (Ar**C**H_3_), 40.6 (**C**H_2_NH), 60.8 (**C**H_2_OH), 70.3 (**C**H_2_OAr), 103.6 (Ar**C**), 113.9 (Ar**C**), 129.5 (Ar**C**), 132.0 (Ar**C**), 133.1 (Ar**C**), 133.7 (Ar**C**), 134.0 (Ar**C**), 144.5 (Ar**C**), 144.7 (Ar**C**), 149.0 (Ar**C**), 152.6 (Ar**C**), 156.5 (Ar**C**), 158.2 (Ar**C**); IR (ATR, cm^−1^): 3326, 2961, 2903, 1610, 1567, 1488, 1452, 1202, 1080, 1041, 972, 818; MS (ESI): *m*/*z* calculated for C_38_H_51_N_4_O_3_: 611.3956 [*M*+H]^+^, found 611.3961; *R_f_* = 0.46 [Al_2_O_3_, CHCl_3_/Et_2_O 1:6 (*v*/*v*)].

## 6. Refinement

Crystal data, data collection and structure refinement details are summarized in Table 3 ▸. The non-hydrogen atoms were refined anisotropically. The positions of the N—H and O—H hydrogen atoms were extracted from difference-Fourier maps. All other hydrogen atoms were positioned geometrically and refined isotropically using a riding model with C—H = 0.95–0.99 Å (alk­yl), 0.95 Å (ar­yl); *U*_iso_(H) = 1.2–1.5*U*_eq_(C).

## Supplementary Material

Crystal structure: contains datablock(s) 1a, 1b. DOI: 10.1107/S2056989023009155/ex2075sup1.cif

Structure factors: contains datablock(s) 1a. DOI: 10.1107/S2056989023009155/ex20751asup2.hkl

Structure factors: contains datablock(s) 1b. DOI: 10.1107/S2056989023009155/ex20751bsup3.hkl

CCDC references: 2301884, 2301883

Additional supporting information:  crystallographic information; 3D view; checkCIF report

## Figures and Tables

**Table 1:** Hydrogen-bond geometry (Å, °) for **1a** [Chem scheme1] *Cg*1, *Cg*2 and *Cg*3 represent the centroids of the C1–C6, C20–C24/N2 and C30–C34/N4 rings, respectively.

*D*—H⋯*A*	*D*—H	H⋯*A*	*D*⋯*A*	*D*—H⋯*A*
N1—H1⋯O3^i^	0.88 (1)	2.46 (2)	3.183 (2)	140 (2)
N1*A*—H2*B*⋯N4^ii^	0.89 (1)	2.12 (1)	3.000 (3)	170 (3)
N1*A*—H1*B*⋯O1*A* ^iii^	0.90 (1)	2.01 (1)	2.893 (3)	167 (3)
O2—H2⋯O3^iv^	0.84	2.26	2.968 (3)	142
O3—H3*A*⋯N2^v^	0.85 (1)	2.00 (1)	2.822 (2)	162 (2)
C10—H10⋯O2^vi^	0.95	2.53	3.418 (4)	156
C12—H12⋯O3	0.95	2.42	2.788 (3)	103
C14—H14*A*⋯O1	0.99	2.47	2.894 (3)	105
C15—H15*C*⋯O1^iv^	0.98	2.52	3.412 (3)	151
C22—H22⋯O1*A* ^vii^	0.95	2.63	3.523 (3)	157
C29—H29*B*⋯N1*A* ^ii^	0.99	2.59	3.374 (3)	137
C37—H37*A*⋯O1	0.99	2.49	3.238 (3)	132
C26—H26*A*⋯*Cg*1^viii^	0.98	2.71	3.658 (3)	164
C18—H18*B*⋯*Cg*2^v^	0.98	2.76	3.679 (3)	156
C38—H38*B*⋯*Cg*3^v^	0.98	2.81	3.545 (3)	132

Symmetry codes: (i) 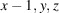 ; (ii) 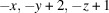 ; (iii) 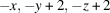 ; (iv) 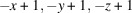 ; (v) 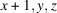 ; (vi) 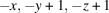 ; (vii) 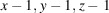 ; (viii) 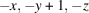 .

**Table 2:** Hydrogen-bond geometry (Å, °) for **1b** [Chem scheme1] *Cg*1, *Cg*2 and *Cg*3 represent the centroids of the C1–C6, C8–C13 and C20–C24/N2 rings, respectively.

*D*—H⋯*A*	*D*—H	H⋯*A*	*D*⋯*A*	*D*—H⋯*A*
N1—H1*B*⋯O3^i^	0.88 (1)	2.21 (1)	3.0704 (18)	165 (2)
O2—H2⋯O1*A*	0.85 (1)	1.86 (2)	2.702 (2)	171 (2)
O2—H2⋯O1*W*	0.85 (1)	1.97 (2)	2.773 (6)	158 (2)
O3—H3⋯O2^ii^	0.85 (1)	1.92 (2)	2.7646 (15)	171 (2)
O1*A*—H1*A*⋯N2^iii^	0.85 (1)	1.97 (2)	2.813 (2)	177 (2)
C10—H10⋯O2	0.95	2.44	2.7925 (17)	102
C12—H12⋯O3	0.95	2.49	2.8362 (17)	101
C24—H24⋯O3^i^	0.95	2.65	3.4259 (19)	139
C27—H27*A*⋯N3	0.99	2.55	3.257 (3)	128
C34*B*—H34*B*⋯O1*W*	0.95	2.16	2.969 (9)	143
C37—H37*A*⋯O1	0.99	2.41	3.1687 (17)	133
C14—H14*B*⋯*Cg*1	0.99	2.88	3.8427 (18)	166
C25—H25*B*⋯*Cg*2^iv^	0.98	2.72	3.4520 (18)	132
C18—H18*B*⋯*Cg*3^ii^	0.98	2.69	3.6317 (16)	161

Symmetry codes: (i) 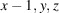 ; (ii) 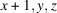 ; (iii) 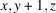 ; (iv) 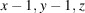 .

**Table 3:** Experimental details

	**1a**	**1b**
Crystal data		
Chemical formula	C_38_H_50_N_4_O_3_·CH_3_NO	C_38_H_50_N_4_O_3_·0.777C_3_H_8_O·0.223H_2_O
*M*_r_	655.86	661.08
Crystal system, space group	Triclinic, *P* 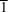	Triclinic, *P* 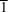
Temperature (K)	100	100
*a*, *b*, *c* (Å)	8.4178 (4), 13.1915 (7), 16.4645 (9)	8.6241 (2), 11.1755 (2), 20.1270 (4)
α, β, γ (°)	91.823 (3), 93.269 (2), 104.534 (2)	102.2675 (12), 98.8911 (10), 92.9034 (10)
*V* (Å^3^)	1764.88 (16)	1865.73 (7)
*Z*	2	2
Radiation type	Mo *K*α	Mo *K*α
μ (mm^−1^)	0.08	0.08
Crystal size (mm)	0.47 × 0.15 × 0.04	0.35 × 0.12 × 0.10

Data collection		
Diffractometer	Bruker Kappa APEXII CCD area detector	Bruker Kappa APEXII CCD area detector
No. of measured, independent and observed [*I* > 2σ(*I*)] reflections	28897, 7558, 5322	32606, 8447, 6498
*R*_int_	0.033	0.029
(sin θ/λ)_max_ (Å^−1^)	0.639	0.647

Refinement		
*R*[*F* ^2^ > 2σ(*F* ^2^)], *wR*(*F* ^2^), *S*	0.059, 0.176, 1.03	0.045, 0.119, 1.03
No. of reflections	7558	8447
No. of parameters	462	526
No. of restraints	7	25
H-atom treatment	H atoms treated by a mixture of independent and constrained refinement	H atoms treated by a mixture of independent and constrained refinement
Δρ_max_, Δρ_min_ (e Å^−3^)	0.54, −0.56	0.33, −0.26

Computer programs: *APEX2* and *SAINT* (Bruker, 2014 ▸), *SHELXS97* (Sheldrick, 2008 ▸), *SHELXL2014/7* (Sheldrick, 2015 ▸), *ORTEP-3 for Windows* (Farrugia, 2012 ▸) and *SHELXTL* (Sheldrick, 2008 ▸).

## supplementary crystallographic information

### 1-{[2,6-Bis(hydroxymethyl)-4-methylphenoxy]methyl}-3,5-bis{[(4,6-\ dimethylpyridin-2-yl)amino]methyl}-2,4,6-triethylbenzene formamide monosolvate (1a) Crystal data

**Table:**

C_38_H_50_N_4_O_3_·CH_3_NO	*Z* = 2
*M**_r_* = 655.86	*F*(000) = 708
Triclinic, *P*1	*D*_x_ = 1.234 Mg m^−^^3^
*a* = 8.4178 (4) Å	Mo *K*α radiation, λ = 0.71073 Å
*b* = 13.1915 (7) Å	Cell parameters from 7296 reflections
*c* = 16.4645 (9) Å	θ = 2.5–27.5°
α = 91.823 (3)°	µ = 0.08 mm^−^^1^
β = 93.269 (2)°	*T* = 100 K
γ = 104.534 (2)°	Plate, colourless
*V* = 1764.88 (16) Å^3^	0.47 × 0.15 × 0.04 mm

### 1-{[2,6-Bis(hydroxymethyl)-4-methylphenoxy]methyl}-3,5-bis{[(4,6-\ dimethylpyridin-2-yl)amino]methyl}-2,4,6-triethylbenzene formamide monosolvate (1a) Data collection

**Table:**

Bruker Kappa APEXII CCD area detector diffractometer	*R*_int_ = 0.033
φ and ω scans	θ_max_ = 27.0°, θ_min_ = 2.0°
28897 measured reflections	*h* = −10→8
7558 independent reflections	*k* = −16→16
5322 reflections with *I* > 2σ(*I*)	*l* = −21→20

### 1-{[2,6-Bis(hydroxymethyl)-4-methylphenoxy]methyl}-3,5-bis{[(4,6-\ dimethylpyridin-2-yl)amino]methyl}-2,4,6-triethylbenzene formamide monosolvate (1a) Refinement

**Table:**

Refinement on *F*^2^	7 restraints
Least-squares matrix: full	Hydrogen site location: mixed
*R*[*F*^2^ > 2σ(*F*^2^)] = 0.059	H atoms treated by a mixture of independent and constrained refinement
*wR*(*F*^2^) = 0.176	*w* = 1/[σ^2^(*F*_o_^2^) + (0.0863*P*)^2^ + 1.3634*P*] where *P* = (*F*_o_^2^ + 2*F*_c_^2^)/3
*S* = 1.03	(Δ/σ)_max_ = 0.001
7558 reflections	Δρ_max_ = 0.54 e Å^−^^3^
462 parameters	Δρ_min_ = −0.56 e Å^−^^3^

### 1-{[2,6-Bis(hydroxymethyl)-4-methylphenoxy]methyl}-3,5-bis{[(4,6-\ dimethylpyridin-2-yl)amino]methyl}-2,4,6-triethylbenzene formamide monosolvate (1a) Special details

**Table:**

Geometry. All esds (except the esd in the dihedral angle between two l.s. planes) are estimated using the full covariance matrix. The cell esds are taken into account individually in the estimation of esds in distances, angles and torsion angles; correlations between esds in cell parameters are only used when they are defined by crystal symmetry. An approximate (isotropic) treatment of cell esds is used for estimating esds involving l.s. planes.

### 1-{[2,6-Bis(hydroxymethyl)-4-methylphenoxy]methyl}-3,5-bis{[(4,6-\ dimethylpyridin-2-yl)amino]methyl}-2,4,6-triethylbenzene formamide monosolvate (1a) Fractional atomic coordinates and isotropic or equivalent isotropic displacement parameters (Å^2^)

**Table:**

	*x*	*y*	*z*	*U*_iso_*/*U*_eq_	
O1	0.54313 (18)	0.67784 (12)	0.35427 (9)	0.0237 (4)	
O2	0.1283 (3)	0.6456 (2)	0.48161 (13)	0.0555 (6)	
H2	0.1915	0.6472	0.5233	0.083*	
O3	0.7900 (2)	0.43445 (14)	0.35900 (10)	0.0310 (4)	
H3A	0.734 (3)	0.393 (2)	0.3212 (14)	0.051 (10)*	
N1	−0.1265 (2)	0.45208 (15)	0.17303 (11)	0.0218 (4)	
H1	−0.095 (3)	0.462 (2)	0.2253 (7)	0.038 (8)*	
N2	−0.3468 (2)	0.32570 (14)	0.21082 (11)	0.0193 (4)	
N3	−0.0035 (3)	0.87558 (16)	0.28947 (12)	0.0281 (5)	
H3	−0.016 (3)	0.8197 (13)	0.3185 (14)	0.028 (7)*	
N4	−0.0200 (2)	1.04735 (15)	0.27966 (12)	0.0270 (4)	
C1	0.3612 (2)	0.68707 (17)	0.23234 (12)	0.0174 (4)	
C2	0.2581 (2)	0.59759 (16)	0.19184 (12)	0.0162 (4)	
C3	0.1025 (2)	0.60250 (16)	0.15845 (12)	0.0167 (4)	
C4	0.0498 (2)	0.69473 (16)	0.16714 (12)	0.0167 (4)	
C5	0.1535 (2)	0.78308 (16)	0.20897 (12)	0.0175 (4)	
C6	0.3090 (2)	0.77953 (16)	0.24189 (12)	0.0178 (4)	
C7	0.5314 (3)	0.68647 (18)	0.26685 (12)	0.0215 (5)	
H7A	0.5644	0.6270	0.2407	0.026*	
H7B	0.6100	0.7519	0.2528	0.026*	
C8	0.4645 (3)	0.57993 (18)	0.37933 (13)	0.0230 (5)	
C9	0.3042 (3)	0.5618 (2)	0.40534 (13)	0.0262 (5)	
C10	0.2286 (3)	0.4623 (2)	0.42953 (14)	0.0294 (5)	
H10	0.1182	0.4482	0.4446	0.035*	
C11	0.3097 (3)	0.38335 (19)	0.43232 (13)	0.0279 (5)	
C12	0.4719 (3)	0.40544 (19)	0.40964 (13)	0.0263 (5)	
H12	0.5302	0.3526	0.4132	0.032*	
C13	0.5498 (3)	0.50207 (19)	0.38218 (13)	0.0239 (5)	
C14	0.2172 (3)	0.6489 (2)	0.41207 (15)	0.0347 (6)	
H14A	0.2998	0.7173	0.4128	0.042*	
H14B	0.1410	0.6444	0.3632	0.042*	
C15	0.2325 (3)	0.2777 (2)	0.46305 (15)	0.0354 (6)	
H15A	0.2252	0.2237	0.4198	0.053*	
H15B	0.1218	0.2763	0.4795	0.053*	
H15C	0.2998	0.2642	0.5100	0.053*	
C16	0.7268 (3)	0.52486 (19)	0.35924 (14)	0.0273 (5)	
H16A	0.7340	0.5537	0.3044	0.033*	
H16B	0.7960	0.5789	0.3983	0.033*	
C17	0.3120 (3)	0.49654 (17)	0.18149 (13)	0.0209 (5)	
H17A	0.2143	0.4362	0.1807	0.025*	
H17B	0.3868	0.4908	0.2288	0.025*	
C18	0.3997 (3)	0.49125 (19)	0.10337 (14)	0.0247 (5)	
H18A	0.3255	0.4957	0.0563	0.037*	
H18B	0.4316	0.4249	0.0993	0.037*	
H18C	0.4981	0.5498	0.1044	0.037*	
C19	−0.0131 (2)	0.50690 (16)	0.11589 (13)	0.0183 (4)	
H19A	−0.0756	0.5283	0.0696	0.022*	
H19B	0.0506	0.4596	0.0942	0.022*	
C20	−0.2538 (2)	0.36779 (16)	0.15051 (13)	0.0167 (4)	
C21	−0.4753 (3)	0.24234 (17)	0.19192 (13)	0.0208 (5)	
C22	−0.5143 (3)	0.19955 (17)	0.11355 (13)	0.0219 (5)	
H22	−0.6065	0.1413	0.1022	0.026*	
C23	−0.4173 (3)	0.24257 (17)	0.05101 (13)	0.0202 (4)	
C24	−0.2857 (2)	0.32763 (16)	0.07005 (13)	0.0182 (4)	
H24	−0.2174	0.3587	0.0289	0.022*	
C25	−0.5729 (3)	0.19805 (19)	0.26176 (15)	0.0304 (5)	
H25A	−0.5021	0.1729	0.3014	0.046*	
H25B	−0.6651	0.1396	0.2416	0.046*	
H25C	−0.6153	0.2527	0.2880	0.046*	
C26	−0.4546 (3)	0.19674 (19)	−0.03442 (14)	0.0275 (5)	
H26A	−0.3748	0.2370	−0.0699	0.041*	
H26B	−0.5658	0.1995	−0.0535	0.041*	
H26C	−0.4476	0.1237	−0.0358	0.041*	
C27	−0.1171 (3)	0.69977 (18)	0.12940 (13)	0.0213 (5)	
H27A	−0.1603	0.7494	0.1626	0.026*	
H27B	−0.1948	0.6297	0.1301	0.026*	
C28	−0.1077 (3)	0.73484 (19)	0.04167 (14)	0.0263 (5)	
H28A	−0.0351	0.8056	0.0410	0.039*	
H28B	−0.2179	0.7350	0.0192	0.039*	
H28C	−0.0639	0.6863	0.0086	0.039*	
C29	0.0948 (3)	0.88172 (17)	0.21910 (13)	0.0204 (4)	
H29A	0.1907	0.9434	0.2265	0.024*	
H29B	0.0281	0.8904	0.1695	0.024*	
C30	−0.0528 (3)	0.9600 (2)	0.32049 (14)	0.0264 (5)	
C31	−0.0658 (3)	1.1312 (2)	0.31098 (17)	0.0372 (6)	
C32	−0.1463 (3)	1.1274 (3)	0.38095 (19)	0.0444 (8)	
H32	−0.1788	1.1873	0.4003	0.053*	
C33	−0.1807 (3)	1.0367 (3)	0.42372 (17)	0.0425 (7)	
C34	−0.1337 (3)	0.9510 (2)	0.39325 (15)	0.0359 (6)	
H34	−0.1555	0.8873	0.4208	0.043*	
C35	−0.0207 (4)	1.2289 (2)	0.2649 (2)	0.0538 (9)	
H35A	−0.0903	1.2202	0.2141	0.081*	
H35B	0.0950	1.2427	0.2523	0.081*	
H35C	−0.0371	1.2880	0.2980	0.081*	
C36	−0.2652 (4)	1.0315 (4)	0.5024 (2)	0.0715 (12)	
H36A	−0.1915	1.0768	0.5444	0.107*	
H36B	−0.2928	0.9591	0.5196	0.107*	
H36C	−0.3660	1.0553	0.4941	0.107*	
C37	0.4222 (3)	0.87555 (17)	0.28621 (13)	0.0224 (5)	
H37A	0.4937	0.8529	0.3280	0.027*	
H37B	0.3552	0.9158	0.3144	0.027*	
C38	0.5300 (3)	0.94671 (18)	0.22818 (15)	0.0278 (5)	
H38A	0.6006	0.9083	0.2021	0.042*	
H38B	0.5986	1.0086	0.2589	0.042*	
H38C	0.4598	0.9687	0.1864	0.042*	
O1A	0.2093 (2)	0.97499 (15)	1.01977 (12)	0.0404 (5)	
N1A	0.0472 (3)	0.95521 (19)	0.90290 (15)	0.0394 (6)	
H1B	−0.028 (3)	0.972 (3)	0.9342 (17)	0.060 (10)*	
H2B	0.042 (4)	0.947 (3)	0.8488 (7)	0.057 (10)*	
C1A	0.1848 (3)	0.9560 (2)	0.94526 (17)	0.0359 (6)	
H1A	0.2716	0.9408	0.9167	0.043*	

### 1-{[2,6-Bis(hydroxymethyl)-4-methylphenoxy]methyl}-3,5-bis{[(4,6-\ dimethylpyridin-2-yl)amino]methyl}-2,4,6-triethylbenzene formamide monosolvate (1a) Atomic displacement parameters (Å^2^)

**Table:**

	*U*^11^	*U*^22^	*U*^33^	*U*^12^	*U*^13^	*U*^23^
O1	0.0235 (8)	0.0289 (9)	0.0183 (8)	0.0069 (7)	−0.0021 (6)	−0.0020 (6)
O2	0.0670 (15)	0.0753 (16)	0.0407 (12)	0.0431 (13)	0.0230 (10)	0.0166 (11)
O3	0.0297 (9)	0.0378 (10)	0.0294 (9)	0.0174 (8)	−0.0024 (7)	−0.0031 (8)
N1	0.0213 (9)	0.0211 (10)	0.0189 (9)	−0.0018 (7)	0.0015 (7)	0.0011 (8)
N2	0.0181 (9)	0.0165 (9)	0.0227 (9)	0.0030 (7)	0.0032 (7)	−0.0010 (7)
N3	0.0365 (11)	0.0266 (11)	0.0261 (10)	0.0146 (9)	0.0125 (8)	0.0037 (9)
N4	0.0255 (10)	0.0247 (11)	0.0323 (11)	0.0118 (8)	−0.0054 (8)	−0.0074 (8)
C1	0.0168 (10)	0.0214 (11)	0.0134 (9)	0.0036 (8)	0.0021 (7)	0.0018 (8)
C2	0.0174 (10)	0.0178 (11)	0.0144 (10)	0.0051 (8)	0.0052 (7)	0.0030 (8)
C3	0.0164 (10)	0.0170 (11)	0.0159 (10)	0.0023 (8)	0.0030 (7)	0.0010 (8)
C4	0.0143 (9)	0.0197 (11)	0.0169 (10)	0.0049 (8)	0.0037 (7)	0.0025 (8)
C5	0.0201 (10)	0.0161 (11)	0.0176 (10)	0.0062 (8)	0.0043 (8)	0.0014 (8)
C6	0.0200 (10)	0.0186 (11)	0.0143 (10)	0.0035 (8)	0.0030 (8)	0.0010 (8)
C7	0.0198 (10)	0.0281 (12)	0.0178 (10)	0.0084 (9)	0.0008 (8)	0.0009 (9)
C8	0.0210 (11)	0.0299 (13)	0.0175 (11)	0.0062 (9)	−0.0011 (8)	−0.0029 (9)
C9	0.0228 (11)	0.0371 (14)	0.0183 (11)	0.0079 (10)	−0.0010 (8)	−0.0003 (10)
C10	0.0212 (11)	0.0423 (15)	0.0225 (12)	0.0049 (10)	−0.0005 (9)	−0.0015 (10)
C11	0.0318 (13)	0.0307 (13)	0.0171 (11)	0.0022 (10)	−0.0039 (9)	−0.0060 (9)
C12	0.0322 (12)	0.0294 (13)	0.0176 (11)	0.0104 (10)	−0.0043 (9)	−0.0057 (9)
C13	0.0238 (11)	0.0320 (13)	0.0157 (10)	0.0075 (10)	−0.0018 (8)	−0.0015 (9)
C14	0.0307 (13)	0.0476 (17)	0.0311 (13)	0.0180 (12)	0.0085 (10)	0.0034 (12)
C15	0.0367 (14)	0.0358 (15)	0.0282 (13)	0.0004 (11)	−0.0017 (10)	−0.0022 (11)
C16	0.0269 (12)	0.0319 (14)	0.0254 (12)	0.0122 (10)	0.0010 (9)	−0.0014 (10)
C17	0.0221 (11)	0.0182 (11)	0.0234 (11)	0.0074 (9)	−0.0003 (8)	0.0004 (9)
C18	0.0228 (11)	0.0261 (12)	0.0280 (12)	0.0119 (9)	0.0007 (9)	−0.0044 (10)
C19	0.0157 (10)	0.0168 (11)	0.0215 (11)	0.0021 (8)	0.0024 (8)	0.0012 (8)
C20	0.0143 (9)	0.0138 (10)	0.0228 (11)	0.0052 (8)	0.0009 (8)	0.0022 (8)
C21	0.0188 (10)	0.0177 (11)	0.0259 (11)	0.0041 (8)	0.0045 (8)	0.0005 (9)
C22	0.0183 (10)	0.0183 (11)	0.0280 (12)	0.0030 (8)	0.0025 (8)	−0.0012 (9)
C23	0.0218 (11)	0.0183 (11)	0.0224 (11)	0.0092 (9)	−0.0001 (8)	−0.0005 (9)
C24	0.0163 (10)	0.0182 (11)	0.0209 (11)	0.0049 (8)	0.0024 (8)	0.0034 (8)
C25	0.0300 (13)	0.0257 (13)	0.0309 (13)	−0.0029 (10)	0.0112 (10)	−0.0025 (10)
C26	0.0295 (12)	0.0291 (13)	0.0231 (12)	0.0066 (10)	−0.0002 (9)	−0.0028 (10)
C27	0.0159 (10)	0.0227 (12)	0.0264 (11)	0.0069 (9)	0.0018 (8)	−0.0003 (9)
C28	0.0250 (12)	0.0269 (13)	0.0278 (12)	0.0100 (10)	−0.0049 (9)	0.0001 (10)
C29	0.0224 (11)	0.0199 (11)	0.0204 (11)	0.0080 (9)	0.0040 (8)	−0.0014 (9)
C30	0.0205 (11)	0.0354 (14)	0.0241 (12)	0.0105 (10)	−0.0024 (9)	−0.0099 (10)
C31	0.0256 (13)	0.0350 (15)	0.0510 (16)	0.0146 (11)	−0.0156 (11)	−0.0192 (12)
C32	0.0279 (14)	0.0510 (19)	0.0564 (18)	0.0209 (13)	−0.0111 (12)	−0.0310 (15)
C33	0.0236 (13)	0.071 (2)	0.0338 (14)	0.0192 (13)	−0.0053 (10)	−0.0261 (14)
C34	0.0287 (13)	0.0533 (17)	0.0278 (13)	0.0149 (12)	0.0029 (10)	−0.0057 (12)
C35	0.0496 (18)	0.0297 (16)	0.085 (2)	0.0216 (14)	−0.0150 (16)	−0.0088 (15)
C36	0.0461 (19)	0.128 (4)	0.0462 (19)	0.037 (2)	0.0071 (15)	−0.033 (2)
C37	0.0225 (11)	0.0223 (12)	0.0210 (11)	0.0041 (9)	−0.0010 (8)	−0.0052 (9)
C38	0.0266 (12)	0.0217 (12)	0.0313 (13)	0.0000 (9)	0.0004 (9)	−0.0044 (10)
O1A	0.0359 (10)	0.0399 (11)	0.0449 (12)	0.0089 (8)	0.0008 (8)	0.0031 (9)
N1A	0.0491 (14)	0.0412 (14)	0.0344 (13)	0.0221 (11)	0.0066 (11)	0.0081 (11)
C1A	0.0381 (15)	0.0267 (14)	0.0449 (16)	0.0093 (11)	0.0109 (12)	0.0082 (12)

### 1-{[2,6-Bis(hydroxymethyl)-4-methylphenoxy]methyl}-3,5-bis{[(4,6-\ dimethylpyridin-2-yl)amino]methyl}-2,4,6-triethylbenzene formamide monosolvate (1a) Geometric parameters (Å, º)

**Table:**

O1—C8	1.383 (3)	C18—H18B	0.9800
O1—C7	1.447 (2)	C18—H18C	0.9800
O2—C14	1.399 (3)	C19—H19A	0.9900
O2—H2	0.8400	C19—H19B	0.9900
O3—C16	1.423 (3)	C20—C24	1.397 (3)
O3—H3A	0.847 (10)	C21—C22	1.380 (3)
N1—C20	1.361 (3)	C21—C25	1.499 (3)
N1—C19	1.455 (3)	C22—C23	1.397 (3)
N1—H1	0.883 (10)	C22—H22	0.9500
N2—C20	1.346 (3)	C23—C24	1.378 (3)
N2—C21	1.349 (3)	C23—C26	1.498 (3)
N3—C30	1.376 (3)	C24—H24	0.9500
N3—C29	1.455 (3)	C25—H25A	0.9800
N3—H3	0.879 (10)	C25—H25B	0.9800
N4—C30	1.329 (3)	C25—H25C	0.9800
N4—C31	1.355 (3)	C26—H26A	0.9800
C1—C2	1.401 (3)	C26—H26B	0.9800
C1—C6	1.404 (3)	C26—H26C	0.9800
C1—C7	1.513 (3)	C27—C28	1.531 (3)
C2—C3	1.409 (3)	C27—H27A	0.9900
C2—C17	1.520 (3)	C27—H27B	0.9900
C3—C4	1.401 (3)	C28—H28A	0.9800
C3—C19	1.510 (3)	C28—H28B	0.9800
C4—C5	1.401 (3)	C28—H28C	0.9800
C4—C27	1.521 (3)	C29—H29A	0.9900
C5—C6	1.401 (3)	C29—H29B	0.9900
C5—C29	1.511 (3)	C30—C34	1.405 (3)
C6—C37	1.517 (3)	C31—C32	1.365 (4)
C7—H7A	0.9900	C31—C35	1.494 (4)
C7—H7B	0.9900	C32—C33	1.384 (5)
C8—C13	1.394 (3)	C32—H32	0.9500
C8—C9	1.404 (3)	C33—C34	1.377 (4)
C9—C10	1.388 (4)	C33—C36	1.508 (4)
C9—C14	1.515 (3)	C34—H34	0.9500
C10—C11	1.382 (4)	C35—H35A	0.9800
C10—H10	0.9500	C35—H35B	0.9800
C11—C12	1.398 (3)	C35—H35C	0.9800
C11—C15	1.499 (4)	C36—H36A	0.9800
C12—C13	1.382 (3)	C36—H36B	0.9800
C12—H12	0.9500	C36—H36C	0.9800
C13—C16	1.517 (3)	C37—C38	1.532 (3)
C14—H14A	0.9900	C37—H37A	0.9900
C14—H14B	0.9900	C37—H37B	0.9900
C15—H15A	0.9800	C38—H38A	0.9800
C15—H15B	0.9800	C38—H38B	0.9800
C15—H15C	0.9800	C38—H38C	0.9800
C16—H16A	0.9900	O1A—C1A	1.239 (3)
C16—H16B	0.9900	N1A—C1A	1.315 (4)
C17—C18	1.527 (3)	N1A—H1B	0.901 (10)
C17—H17A	0.9900	N1A—H2B	0.890 (10)
C17—H17B	0.9900	C1A—H1A	0.9500
C18—H18A	0.9800		
			
C8—O1—C7	113.60 (16)	N2—C20—C24	122.24 (19)
C14—O2—H2	109.5	N1—C20—C24	122.13 (19)
C16—O3—H3A	106 (2)	N2—C21—C22	122.4 (2)
C20—N1—C19	123.01 (18)	N2—C21—C25	115.76 (19)
C20—N1—H1	117.4 (19)	C22—C21—C25	121.8 (2)
C19—N1—H1	117.1 (19)	C21—C22—C23	119.5 (2)
C20—N2—C21	118.20 (18)	C21—C22—H22	120.3
C30—N3—C29	122.7 (2)	C23—C22—H22	120.3
C30—N3—H3	118.3 (17)	C24—C23—C22	118.3 (2)
C29—N3—H3	118.1 (17)	C24—C23—C26	120.9 (2)
C30—N4—C31	117.7 (2)	C22—C23—C26	120.8 (2)
C2—C1—C6	120.73 (18)	C23—C24—C20	119.36 (19)
C2—C1—C7	120.88 (19)	C23—C24—H24	120.3
C6—C1—C7	118.40 (19)	C20—C24—H24	120.3
C1—C2—C3	119.03 (18)	C21—C25—H25A	109.5
C1—C2—C17	121.45 (18)	C21—C25—H25B	109.5
C3—C2—C17	119.50 (18)	H25A—C25—H25B	109.5
C4—C3—C2	120.61 (19)	C21—C25—H25C	109.5
C4—C3—C19	118.92 (18)	H25A—C25—H25C	109.5
C2—C3—C19	120.41 (18)	H25B—C25—H25C	109.5
C3—C4—C5	119.65 (18)	C23—C26—H26A	109.5
C3—C4—C27	120.14 (19)	C23—C26—H26B	109.5
C5—C4—C27	120.19 (18)	H26A—C26—H26B	109.5
C6—C5—C4	120.32 (19)	C23—C26—H26C	109.5
C6—C5—C29	120.37 (19)	H26A—C26—H26C	109.5
C4—C5—C29	119.31 (18)	H26B—C26—H26C	109.5
C5—C6—C1	119.63 (19)	C4—C27—C28	112.15 (17)
C5—C6—C37	120.37 (19)	C4—C27—H27A	109.2
C1—C6—C37	119.98 (18)	C28—C27—H27A	109.2
O1—C7—C1	113.79 (16)	C4—C27—H27B	109.2
O1—C7—H7A	108.8	C28—C27—H27B	109.2
C1—C7—H7A	108.8	H27A—C27—H27B	107.9
O1—C7—H7B	108.8	C27—C28—H28A	109.5
C1—C7—H7B	108.8	C27—C28—H28B	109.5
H7A—C7—H7B	107.7	H28A—C28—H28B	109.5
O1—C8—C13	118.96 (19)	C27—C28—H28C	109.5
O1—C8—C9	120.0 (2)	H28A—C28—H28C	109.5
C13—C8—C9	121.0 (2)	H28B—C28—H28C	109.5
C10—C9—C8	118.4 (2)	N3—C29—C5	109.95 (17)
C10—C9—C14	119.8 (2)	N3—C29—H29A	109.7
C8—C9—C14	121.7 (2)	C5—C29—H29A	109.7
C11—C10—C9	121.8 (2)	N3—C29—H29B	109.7
C11—C10—H10	119.1	C5—C29—H29B	109.7
C9—C10—H10	119.1	H29A—C29—H29B	108.2
C10—C11—C12	118.3 (2)	N4—C30—N3	117.7 (2)
C10—C11—C15	122.1 (2)	N4—C30—C34	123.0 (2)
C12—C11—C15	119.5 (2)	N3—C30—C34	119.4 (2)
C13—C12—C11	121.9 (2)	N4—C31—C32	122.3 (3)
C13—C12—H12	119.1	N4—C31—C35	115.9 (3)
C11—C12—H12	119.1	C32—C31—C35	121.8 (3)
C12—C13—C8	118.5 (2)	C31—C32—C33	120.2 (2)
C12—C13—C16	120.9 (2)	C31—C32—H32	119.9
C8—C13—C16	120.5 (2)	C33—C32—H32	119.9
O2—C14—C9	113.4 (2)	C34—C33—C32	118.3 (3)
O2—C14—H14A	108.9	C34—C33—C36	120.5 (3)
C9—C14—H14A	108.9	C32—C33—C36	121.2 (3)
O2—C14—H14B	108.9	C33—C34—C30	118.5 (3)
C9—C14—H14B	108.9	C33—C34—H34	120.7
H14A—C14—H14B	107.7	C30—C34—H34	120.7
C11—C15—H15A	109.5	C31—C35—H35A	109.5
C11—C15—H15B	109.5	C31—C35—H35B	109.5
H15A—C15—H15B	109.5	H35A—C35—H35B	109.5
C11—C15—H15C	109.5	C31—C35—H35C	109.5
H15A—C15—H15C	109.5	H35A—C35—H35C	109.5
H15B—C15—H15C	109.5	H35B—C35—H35C	109.5
O3—C16—C13	112.9 (2)	C33—C36—H36A	109.5
O3—C16—H16A	109.0	C33—C36—H36B	109.5
C13—C16—H16A	109.0	H36A—C36—H36B	109.5
O3—C16—H16B	109.0	C33—C36—H36C	109.5
C13—C16—H16B	109.0	H36A—C36—H36C	109.5
H16A—C16—H16B	107.8	H36B—C36—H36C	109.5
C2—C17—C18	112.48 (17)	C6—C37—C38	112.11 (18)
C2—C17—H17A	109.1	C6—C37—H37A	109.2
C18—C17—H17A	109.1	C38—C37—H37A	109.2
C2—C17—H17B	109.1	C6—C37—H37B	109.2
C18—C17—H17B	109.1	C38—C37—H37B	109.2
H17A—C17—H17B	107.8	H37A—C37—H37B	107.9
C17—C18—H18A	109.5	C37—C38—H38A	109.5
C17—C18—H18B	109.5	C37—C38—H38B	109.5
H18A—C18—H18B	109.5	H38A—C38—H38B	109.5
C17—C18—H18C	109.5	C37—C38—H38C	109.5
H18A—C18—H18C	109.5	H38A—C38—H38C	109.5
H18B—C18—H18C	109.5	H38B—C38—H38C	109.5
N1—C19—C3	109.64 (17)	C1A—N1A—H1B	112 (2)
N1—C19—H19A	109.7	C1A—N1A—H2B	120 (2)
C3—C19—H19A	109.7	H1B—N1A—H2B	127 (3)
N1—C19—H19B	109.7	O1A—C1A—N1A	124.7 (3)
C3—C19—H19B	109.7	O1A—C1A—H1A	117.6
H19A—C19—H19B	108.2	N1A—C1A—H1A	117.6
N2—C20—N1	115.64 (18)		
			
C6—C1—C2—C3	1.9 (3)	C10—C9—C14—O2	37.5 (3)
C7—C1—C2—C3	−178.07 (18)	C8—C9—C14—O2	−138.9 (2)
C6—C1—C2—C17	−179.53 (18)	C12—C13—C16—O3	7.8 (3)
C7—C1—C2—C17	0.4 (3)	C8—C13—C16—O3	−175.02 (19)
C1—C2—C3—C4	−1.5 (3)	C1—C2—C17—C18	−89.7 (2)
C17—C2—C3—C4	180.00 (18)	C3—C2—C17—C18	88.8 (2)
C1—C2—C3—C19	−178.76 (18)	C20—N1—C19—C3	174.41 (18)
C17—C2—C3—C19	2.7 (3)	C4—C3—C19—N1	−82.5 (2)
C2—C3—C4—C5	0.4 (3)	C2—C3—C19—N1	94.8 (2)
C19—C3—C4—C5	177.79 (18)	C21—N2—C20—N1	179.63 (18)
C2—C3—C4—C27	178.81 (18)	C21—N2—C20—C24	−0.4 (3)
C19—C3—C4—C27	−3.8 (3)	C19—N1—C20—N2	179.68 (18)
C3—C4—C5—C6	0.1 (3)	C19—N1—C20—C24	−0.3 (3)
C27—C4—C5—C6	−178.27 (18)	C20—N2—C21—C22	−0.2 (3)
C3—C4—C5—C29	−179.15 (18)	C20—N2—C21—C25	179.25 (19)
C27—C4—C5—C29	2.5 (3)	N2—C21—C22—C23	0.8 (3)
C4—C5—C6—C1	0.4 (3)	C25—C21—C22—C23	−178.7 (2)
C29—C5—C6—C1	179.62 (18)	C21—C22—C23—C24	−0.6 (3)
C4—C5—C6—C37	179.18 (18)	C21—C22—C23—C26	178.9 (2)
C29—C5—C6—C37	−1.6 (3)	C22—C23—C24—C20	0.0 (3)
C2—C1—C6—C5	−1.4 (3)	C26—C23—C24—C20	−179.49 (19)
C7—C1—C6—C5	178.59 (18)	N2—C20—C24—C23	0.5 (3)
C2—C1—C6—C37	179.78 (18)	N1—C20—C24—C23	−179.53 (19)
C7—C1—C6—C37	−0.2 (3)	C3—C4—C27—C28	−88.6 (2)
C8—O1—C7—C1	71.1 (2)	C5—C4—C27—C28	89.7 (2)
C2—C1—C7—O1	−103.1 (2)	C30—N3—C29—C5	170.2 (2)
C6—C1—C7—O1	76.9 (2)	C6—C5—C29—N3	−94.9 (2)
C7—O1—C8—C13	86.4 (2)	C4—C5—C29—N3	84.3 (2)
C7—O1—C8—C9	−96.8 (2)	C31—N4—C30—N3	−178.8 (2)
O1—C8—C9—C10	179.56 (19)	C31—N4—C30—C34	0.8 (3)
C13—C8—C9—C10	−3.7 (3)	C29—N3—C30—N4	5.8 (3)
O1—C8—C9—C14	−4.0 (3)	C29—N3—C30—C34	−173.8 (2)
C13—C8—C9—C14	172.7 (2)	C30—N4—C31—C32	−1.5 (3)
C8—C9—C10—C11	3.2 (3)	C30—N4—C31—C35	177.7 (2)
C14—C9—C10—C11	−173.3 (2)	N4—C31—C32—C33	1.5 (4)
C9—C10—C11—C12	−0.3 (3)	C35—C31—C32—C33	−177.6 (2)
C9—C10—C11—C15	176.3 (2)	C31—C32—C33—C34	−0.8 (4)
C10—C11—C12—C13	−2.3 (3)	C31—C32—C33—C36	178.3 (3)
C15—C11—C12—C13	−178.9 (2)	C32—C33—C34—C30	0.2 (4)
C11—C12—C13—C8	1.7 (3)	C36—C33—C34—C30	−178.9 (2)
C11—C12—C13—C16	179.0 (2)	N4—C30—C34—C33	−0.2 (4)
O1—C8—C13—C12	178.08 (18)	N3—C30—C34—C33	179.4 (2)
C9—C8—C13—C12	1.3 (3)	C5—C6—C37—C38	−88.2 (2)
O1—C8—C13—C16	0.8 (3)	C1—C6—C37—C38	90.6 (2)
C9—C8—C13—C16	−175.9 (2)		

### 1-{[2,6-Bis(hydroxymethyl)-4-methylphenoxy]methyl}-3,5-bis{[(4,6-\ dimethylpyridin-2-yl)amino]methyl}-2,4,6-triethylbenzene formamide monosolvate (1a) Hydrogen-bond geometry (Å, º)

*Cg*1, *Cg*2 and *Cg*3 represent the centroids of the C1–C6, C20–C24/N2 and C30–C34/N4 rings, respectively.

**Table:**

*D*—H···*A*	*D*—H	H···*A*	*D*···*A*	*D*—H···*A*
N1—H1···O3^i^	0.88 (1)	2.46 (2)	3.183 (2)	140 (2)
N1*A*—H2*B*···N4^ii^	0.89 (1)	2.12 (1)	3.000 (3)	170 (3)
N1*A*—H1*B*···O1*A*^iii^	0.90 (1)	2.01 (1)	2.893 (3)	167 (3)
O2—H2···O3^iv^	0.84	2.26	2.968 (3)	142
O3—H3*A*···N2^v^	0.85 (1)	2.00 (1)	2.822 (2)	162 (2)
C10—H10···O2^vi^	0.95	2.53	3.418 (4)	156
C12—H12···O3	0.95	2.42	2.788 (3)	103
C14—H14*A*···O1	0.99	2.47	2.894 (3)	105
C15—H15*C*···O1^iv^	0.98	2.52	3.412 (3)	151
C22—H22···O1*A*^vii^	0.95	2.63	3.523 (3)	157
C29—H29*B*···N1*A*^ii^	0.99	2.59	3.374 (3)	137
C37—H37*A*···O1	0.99	2.49	3.238 (3)	132
C26—H26*A*···*Cg*1^viii^	0.98	2.71	3.658 (3)	164
C18—H18*B*···*Cg*2^v^	0.98	2.76	3.679 (3)	156
C38—H38*B*···*Cg*3^v^	0.98	2.81	3.545 (3)	132

Symmetry codes: (i) *x*−1, *y*, *z*; (ii) −*x*, −*y*+2, −*z*+1; (iii) −*x*, −*y*+2, −*z*+2; (iv) −*x*+1, −*y*+1, −*z*+1; (v) *x*+1, *y*, *z*; (vi) −*x*, −*y*+1, −*z*+1; (vii) *x*−1, *y*−1, *z*−1; (viii) −*x*, −*y*+1, −*z*.

### 1-{[2,6-Bis(hydroxymethyl)-4-methylphenoxy]methyl}-3,5-bis{[(4,6-dimethylpyridin-2-yl)amino]methyl}-2,4,6-triethylbenzene–n-propanol–water (1/0.777/0.223) (1b) Crystal data

**Table:**

C_38_H_50_N_4_O_3_·0.777C_3_H_8_O·0.223H_2_O	*Z* = 2
*M**_r_* = 661.08	*F*(000) = 716.4
Triclinic, *P*1	*D*_x_ = 1.177 Mg m^−^^3^
*a* = 8.6241 (2) Å	Mo *K*α radiation, λ = 0.71073 Å
*b* = 11.1755 (2) Å	Cell parameters from 9616 reflections
*c* = 20.1270 (4) Å	θ = 2.3–28.5°
α = 102.2675 (12)°	µ = 0.08 mm^−^^1^
β = 98.8911 (10)°	*T* = 100 K
γ = 92.9034 (10)°	Rod, colourless
*V* = 1865.73 (7) Å^3^	0.35 × 0.12 × 0.10 mm

### 1-{[2,6-Bis(hydroxymethyl)-4-methylphenoxy]methyl}-3,5-bis{[(4,6-dimethylpyridin-2-yl)amino]methyl}-2,4,6-triethylbenzene–n-propanol–water (1/0.777/0.223) (1b) Data collection

**Table:**

Bruker Kappa APEXII CCD area detector diffractometer	*R*_int_ = 0.029
φ and ω scans	θ_max_ = 27.4°, θ_min_ = 2.5°
32606 measured reflections	*h* = −11→11
8447 independent reflections	*k* = −14→13
6498 reflections with *I* > 2σ(*I*)	*l* = −26→26

### 1-{[2,6-Bis(hydroxymethyl)-4-methylphenoxy]methyl}-3,5-bis{[(4,6-dimethylpyridin-2-yl)amino]methyl}-2,4,6-triethylbenzene–n-propanol–water (1/0.777/0.223) (1b) Refinement

**Table:**

Refinement on *F*^2^	25 restraints
Least-squares matrix: full	Hydrogen site location: mixed
*R*[*F*^2^ > 2σ(*F*^2^)] = 0.045	H atoms treated by a mixture of independent and constrained refinement
*wR*(*F*^2^) = 0.119	*w* = 1/[σ^2^(*F*_o_^2^) + (0.0511*P*)^2^ + 0.8172*P*] where *P* = (*F*_o_^2^ + 2*F*_c_^2^)/3
*S* = 1.03	(Δ/σ)_max_ = 0.001
8447 reflections	Δρ_max_ = 0.33 e Å^−^^3^
526 parameters	Δρ_min_ = −0.26 e Å^−^^3^

### 1-{[2,6-Bis(hydroxymethyl)-4-methylphenoxy]methyl}-3,5-bis{[(4,6-dimethylpyridin-2-yl)amino]methyl}-2,4,6-triethylbenzene–n-propanol–water (1/0.777/0.223) (1b) Special details

**Table:**

Geometry. All esds (except the esd in the dihedral angle between two l.s. planes) are estimated using the full covariance matrix. The cell esds are taken into account individually in the estimation of esds in distances, angles and torsion angles; correlations between esds in cell parameters are only used when they are defined by crystal symmetry. An approximate (isotropic) treatment of cell esds is used for estimating esds involving l.s. planes.

### 1-{[2,6-Bis(hydroxymethyl)-4-methylphenoxy]methyl}-3,5-bis{[(4,6-dimethylpyridin-2-yl)amino]methyl}-2,4,6-triethylbenzene–n-propanol–water (1/0.777/0.223) (1b) Fractional atomic coordinates and isotropic or equivalent isotropic displacement parameters (Å^2^)

**Table:**

	*x*	*y*	*z*	*U*_iso_*/*U*_eq_	Occ. (<1)
O1	1.09845 (11)	0.21196 (9)	0.25578 (5)	0.0186 (2)	
O2	0.73337 (11)	0.35817 (9)	0.35632 (5)	0.0202 (2)	
H2	0.698 (2)	0.4167 (15)	0.3391 (10)	0.051 (6)*	
O3	1.53531 (11)	0.16740 (10)	0.37193 (6)	0.0228 (2)	
H3	1.595 (2)	0.2298 (14)	0.3714 (11)	0.055 (7)*	
N1	0.56482 (15)	−0.11050 (12)	0.33365 (7)	0.0224 (3)	
H1B	0.574 (2)	−0.0295 (9)	0.3422 (9)	0.029 (5)*	
N2	0.45201 (13)	−0.28824 (11)	0.35529 (6)	0.0169 (2)	
C1	0.89520 (15)	0.03615 (13)	0.20963 (7)	0.0172 (3)	
C2	0.84608 (15)	−0.04442 (13)	0.24865 (7)	0.0168 (3)	
C3	0.68766 (16)	−0.09218 (13)	0.23522 (7)	0.0177 (3)	
C4	0.57876 (16)	−0.05761 (14)	0.18474 (7)	0.0194 (3)	
C5	0.62879 (16)	0.02517 (14)	0.14751 (7)	0.0194 (3)	
C6	0.78643 (16)	0.07368 (13)	0.16017 (7)	0.0181 (3)	
C7	1.06643 (15)	0.08464 (13)	0.21945 (7)	0.0188 (3)	
H7A	1.1318	0.0338	0.2453	0.023*	
H7B	1.0984	0.0754	0.1736	0.023*	
C8	1.11557 (15)	0.23328 (12)	0.32727 (7)	0.0150 (3)	
C9	0.99458 (15)	0.28480 (13)	0.35935 (7)	0.0170 (3)	
C10	1.01731 (15)	0.31362 (13)	0.43082 (7)	0.0181 (3)	
H10	0.9363	0.3494	0.4534	0.022*	
C11	1.15576 (16)	0.29156 (13)	0.47053 (7)	0.0189 (3)	
C12	1.27600 (15)	0.24325 (13)	0.43672 (7)	0.0183 (3)	
H12	1.3717	0.2293	0.4632	0.022*	
C13	1.25877 (15)	0.21523 (12)	0.36541 (7)	0.0164 (3)	
C14	0.84727 (17)	0.31143 (16)	0.31560 (8)	0.0272 (3)	
H14A	0.8754	0.3721	0.2891	0.033*	
H14B	0.8009	0.2350	0.2822	0.033*	
C15	1.17600 (18)	0.32174 (16)	0.54809 (8)	0.0273 (3)	
H15A	1.1270	0.2537	0.5635	0.041*	
H15B	1.1256	0.3969	0.5637	0.041*	
H15C	1.2885	0.3342	0.5676	0.041*	
C16	1.39215 (16)	0.17201 (15)	0.32754 (8)	0.0246 (3)	
H16A	1.3594	0.0891	0.2980	0.029*	
H16B	1.4108	0.2280	0.2970	0.029*	
C17	0.96211 (16)	−0.08286 (14)	0.30354 (7)	0.0206 (3)	
H17A	0.9064	−0.0985	0.3407	0.025*	
H17B	1.0451	−0.0149	0.3240	0.025*	
C18	1.03917 (17)	−0.19885 (14)	0.27405 (8)	0.0259 (3)	
H18A	0.9576	−0.2670	0.2548	0.039*	
H18B	1.1133	−0.2203	0.3109	0.039*	
H18C	1.0958	−0.1834	0.2377	0.039*	
C19	0.63062 (16)	−0.17910 (13)	0.27645 (7)	0.0190 (3)	
H19A	0.5494	−0.2412	0.2464	0.023*	
H19B	0.7196	−0.2226	0.2942	0.023*	
C20	0.46973 (15)	−0.16490 (13)	0.36898 (7)	0.0164 (3)	
C21	0.35623 (16)	−0.34081 (13)	0.39077 (8)	0.0201 (3)	
C22	0.27879 (16)	−0.27285 (14)	0.43864 (8)	0.0213 (3)	
H22	0.2130	−0.3132	0.4623	0.026*	
C23	0.29668 (15)	−0.14425 (13)	0.45257 (7)	0.0182 (3)	
C24	0.39397 (16)	−0.09037 (13)	0.41748 (7)	0.0179 (3)	
H24	0.4101	−0.0034	0.4258	0.021*	
C25	0.3398 (2)	−0.47858 (15)	0.37504 (10)	0.0357 (4)	
H25A	0.3121	−0.5103	0.3250	0.054*	
H25B	0.2569	−0.5070	0.3977	0.054*	
H25C	0.4398	−0.5086	0.3921	0.054*	
C26	0.20822 (17)	−0.06871 (15)	0.50283 (8)	0.0251 (3)	
H26A	0.2463	0.0182	0.5111	0.038*	
H26B	0.2252	−0.0958	0.5464	0.038*	
H26C	0.0955	−0.0793	0.4837	0.038*	
C27	0.40803 (16)	−0.11206 (16)	0.16904 (8)	0.0275 (3)	
H27A	0.3392	−0.0505	0.1548	0.033*	
H27B	0.3779	−0.1305	0.2115	0.033*	
C28	0.3810 (2)	−0.22925 (19)	0.11221 (10)	0.0407 (4)	
H28A	0.4138	−0.2121	0.0704	0.061*	
H28B	0.2689	−0.2583	0.1023	0.061*	
H28C	0.4430	−0.2926	0.1274	0.061*	
C29	0.50860 (18)	0.06695 (16)	0.09561 (8)	0.0248 (3)	0.7770 (18)
H29A	0.557 (3)	0.092 (2)	0.0586 (10)	0.012 (6)*	0.7770 (18)
H29B	0.435 (6)	−0.003 (4)	0.067 (3)	0.07 (2)*	0.7770 (18)
N3	0.4192 (2)	0.15643 (19)	0.13029 (10)	0.0251 (4)	0.7770 (18)
H3A	0.436 (2)	0.1759 (19)	0.1758 (5)	0.020 (5)*	0.7770 (18)
N4	0.2978 (4)	0.2039 (3)	0.02829 (14)	0.0257 (6)	0.7770 (18)
C30	0.3194 (2)	0.2263 (2)	0.09810 (11)	0.0180 (4)	0.7770 (18)
C31	0.2037 (3)	0.2785 (3)	−0.00194 (12)	0.0248 (5)	0.7770 (18)
C32	0.1361 (3)	0.3734 (3)	0.03600 (12)	0.0298 (6)	0.7770 (18)
H32	0.0745	0.4257	0.0133	0.036*	0.7770 (18)
C33	0.1582 (2)	0.3925 (2)	0.10761 (13)	0.0252 (5)	0.7770 (18)
C34	0.2507 (2)	0.31710 (17)	0.13893 (10)	0.0189 (4)	0.7770 (18)
H34	0.2672	0.3270	0.1876	0.023*	0.7770 (18)
C35	0.1725 (4)	0.2488 (3)	−0.07895 (13)	0.0414 (6)	0.7770 (18)
H35A	0.2727	0.2440	−0.0962	0.062*	0.7770 (18)
H35B	0.1148	0.3132	−0.0953	0.062*	0.7770 (18)
H35C	0.1094	0.1696	−0.0959	0.062*	0.7770 (18)
C36	0.0891 (3)	0.4958 (2)	0.15129 (15)	0.0446 (6)	0.7770 (18)
H36A	0.0779	0.4752	0.1954	0.067*	0.7770 (18)
H36B	−0.0146	0.5081	0.1272	0.067*	0.7770 (18)
H36C	0.1588	0.5714	0.1598	0.067*	0.7770 (18)
C29B	0.50860 (18)	0.06695 (16)	0.09561 (8)	0.0248 (3)	0.2230 (18)
H29C	0.579 (9)	0.122 (7)	0.079 (5)	0.02 (3)*	0.2230 (18)
H29D	0.430 (10)	−0.003 (7)	0.072 (6)	0.00 (3)*	0.2230 (18)
N3B	0.4781 (9)	0.2014 (7)	0.1409 (4)	0.0251 (4)	0.2230 (18)
H3B	0.5311	0.2308	0.1828	0.030*	0.2230 (18)
C30B	0.3666 (9)	0.2663 (7)	0.1110 (4)	0.0180 (4)	0.2230 (18)
N4B	0.3042 (18)	0.2338 (12)	0.0422 (5)	0.0257 (6)	0.2230 (18)
C31B	0.1883 (14)	0.3023 (12)	0.0176 (4)	0.0248 (5)	0.2230 (18)
C32B	0.1411 (13)	0.3978 (10)	0.0642 (5)	0.0298 (6)	0.2230 (18)
H32B	0.0576	0.4426	0.0484	0.036*	0.2230 (18)
C33B	0.2104 (9)	0.4298 (7)	0.1321 (4)	0.0252 (5)	0.2230 (18)
C34B	0.3272 (8)	0.3642 (6)	0.1572 (3)	0.0189 (4)	0.2230 (18)
H34B	0.3780	0.3855	0.2040	0.023*	0.2230 (18)
C35B	0.1166 (13)	0.2733 (10)	−0.0584 (4)	0.0414 (6)	0.2230 (18)
H35D	0.1545	0.3374	−0.0800	0.062*	0.2230 (18)
H35E	0.0016	0.2701	−0.0633	0.062*	0.2230 (18)
H35F	0.1476	0.1936	−0.0810	0.062*	0.2230 (18)
C36B	0.1663 (12)	0.5312 (9)	0.1880 (5)	0.0446 (6)	0.2230 (18)
H36D	0.0909	0.5805	0.1668	0.067*	0.2230 (18)
H36E	0.2612	0.5839	0.2123	0.067*	0.2230 (18)
H36F	0.1185	0.4942	0.2209	0.067*	0.2230 (18)
C37	0.83976 (17)	0.16528 (14)	0.12075 (7)	0.0229 (3)	
H37A	0.9310	0.2190	0.1496	0.028*	
H37B	0.7535	0.2178	0.1116	0.028*	
C38	0.88619 (19)	0.10262 (17)	0.05233 (8)	0.0303 (4)	
H38A	0.9800	0.0588	0.0614	0.045*	
H38B	0.9091	0.1648	0.0266	0.045*	
H38C	0.7992	0.0442	0.0251	0.045*	
O1A	0.64599 (19)	0.54143 (15)	0.29387 (8)	0.0208 (3)	0.7770 (18)
H1A	0.585 (3)	0.5911 (19)	0.3115 (12)	0.040 (7)*	0.7770 (18)
C1A	0.5739 (2)	0.49317 (18)	0.22359 (11)	0.0214 (4)	0.7770 (18)
H1A1	0.4747	0.5319	0.2138	0.026*	0.7770 (18)
H1A2	0.5475	0.4036	0.2163	0.026*	0.7770 (18)
C2A	0.6820 (2)	0.5168 (2)	0.17434 (10)	0.0276 (5)	0.7770 (18)
H2A1	0.7823	0.4801	0.1853	0.033*	0.7770 (18)
H2A2	0.7063	0.6065	0.1812	0.033*	0.7770 (18)
C3A	0.6105 (3)	0.4638 (2)	0.09917 (10)	0.0294 (5)	0.7770 (18)
H3A1	0.5876	0.3748	0.0919	0.044*	0.7770 (18)
H3A2	0.6852	0.4810	0.0696	0.044*	0.7770 (18)
H3A3	0.5128	0.5016	0.0876	0.044*	0.7770 (18)
O1W	0.5762 (7)	0.4957 (6)	0.2720 (3)	0.0296 (13)	0.2230 (18)

### 1-{[2,6-Bis(hydroxymethyl)-4-methylphenoxy]methyl}-3,5-bis{[(4,6-dimethylpyridin-2-yl)amino]methyl}-2,4,6-triethylbenzene–n-propanol–water (1/0.777/0.223) (1b) Atomic displacement parameters (Å^2^)

**Table:**

	*U*^11^	*U*^22^	*U*^33^	*U*^12^	*U*^13^	*U*^23^
O1	0.0204 (5)	0.0201 (5)	0.0178 (5)	0.0030 (4)	0.0064 (4)	0.0072 (4)
O2	0.0156 (5)	0.0217 (5)	0.0265 (5)	0.0072 (4)	0.0071 (4)	0.0086 (4)
O3	0.0127 (4)	0.0220 (6)	0.0356 (6)	0.0032 (4)	0.0032 (4)	0.0107 (5)
N1	0.0283 (6)	0.0178 (7)	0.0258 (7)	0.0030 (5)	0.0147 (5)	0.0079 (5)
N2	0.0161 (5)	0.0168 (6)	0.0190 (6)	0.0021 (4)	0.0033 (4)	0.0064 (5)
C1	0.0161 (6)	0.0197 (7)	0.0173 (7)	0.0049 (5)	0.0061 (5)	0.0044 (6)
C2	0.0176 (6)	0.0197 (7)	0.0151 (6)	0.0070 (5)	0.0054 (5)	0.0052 (5)
C3	0.0188 (6)	0.0210 (7)	0.0169 (7)	0.0066 (5)	0.0074 (5)	0.0078 (6)
C4	0.0153 (6)	0.0249 (8)	0.0211 (7)	0.0067 (5)	0.0067 (5)	0.0081 (6)
C5	0.0188 (6)	0.0251 (8)	0.0183 (7)	0.0098 (6)	0.0064 (5)	0.0094 (6)
C6	0.0205 (6)	0.0207 (7)	0.0161 (7)	0.0062 (5)	0.0074 (5)	0.0066 (6)
C7	0.0166 (6)	0.0223 (7)	0.0188 (7)	0.0039 (5)	0.0058 (5)	0.0048 (6)
C8	0.0160 (6)	0.0127 (6)	0.0184 (7)	−0.0003 (5)	0.0051 (5)	0.0067 (5)
C9	0.0142 (6)	0.0181 (7)	0.0211 (7)	0.0019 (5)	0.0038 (5)	0.0090 (6)
C10	0.0159 (6)	0.0205 (7)	0.0203 (7)	0.0018 (5)	0.0066 (5)	0.0072 (6)
C11	0.0198 (6)	0.0165 (7)	0.0206 (7)	−0.0006 (5)	0.0027 (5)	0.0056 (6)
C12	0.0155 (6)	0.0148 (7)	0.0246 (7)	0.0011 (5)	0.0003 (5)	0.0065 (6)
C13	0.0146 (6)	0.0122 (6)	0.0234 (7)	0.0008 (5)	0.0045 (5)	0.0054 (5)
C14	0.0207 (7)	0.0426 (10)	0.0206 (7)	0.0165 (7)	0.0054 (6)	0.0079 (7)
C15	0.0269 (8)	0.0338 (9)	0.0203 (7)	0.0056 (6)	0.0008 (6)	0.0053 (7)
C16	0.0143 (6)	0.0308 (9)	0.0273 (8)	0.0064 (6)	0.0031 (6)	0.0029 (7)
C17	0.0201 (7)	0.0251 (8)	0.0177 (7)	0.0041 (6)	0.0006 (5)	0.0087 (6)
C18	0.0203 (7)	0.0264 (8)	0.0312 (8)	0.0075 (6)	−0.0015 (6)	0.0101 (7)
C19	0.0192 (6)	0.0218 (7)	0.0200 (7)	0.0049 (5)	0.0076 (5)	0.0095 (6)
C20	0.0147 (6)	0.0198 (7)	0.0162 (6)	0.0011 (5)	0.0018 (5)	0.0079 (5)
C21	0.0184 (6)	0.0186 (7)	0.0254 (7)	0.0012 (5)	0.0044 (5)	0.0090 (6)
C22	0.0188 (6)	0.0236 (8)	0.0254 (8)	−0.0002 (6)	0.0080 (6)	0.0117 (6)
C23	0.0146 (6)	0.0221 (7)	0.0184 (7)	0.0029 (5)	0.0025 (5)	0.0059 (6)
C24	0.0193 (6)	0.0150 (7)	0.0206 (7)	0.0023 (5)	0.0042 (5)	0.0059 (6)
C25	0.0391 (9)	0.0212 (9)	0.0521 (11)	0.0000 (7)	0.0222 (8)	0.0097 (8)
C26	0.0237 (7)	0.0267 (8)	0.0267 (8)	0.0032 (6)	0.0109 (6)	0.0046 (6)
C27	0.0146 (6)	0.0417 (10)	0.0319 (8)	0.0051 (6)	0.0051 (6)	0.0194 (7)
C28	0.0285 (8)	0.0520 (12)	0.0387 (10)	−0.0090 (8)	−0.0030 (7)	0.0130 (9)
C29	0.0224 (7)	0.0351 (9)	0.0230 (8)	0.0132 (7)	0.0067 (6)	0.0154 (7)
N3	0.0277 (11)	0.0317 (13)	0.0181 (9)	0.0151 (8)	0.0019 (8)	0.0086 (9)
N4	0.0223 (7)	0.0286 (18)	0.0277 (14)	0.0028 (12)	0.0026 (10)	0.0109 (10)
C30	0.0127 (10)	0.0196 (12)	0.0242 (10)	−0.0010 (7)	0.0029 (8)	0.0116 (9)
C31	0.0271 (10)	0.0277 (15)	0.0251 (15)	0.0065 (9)	0.0085 (11)	0.0141 (12)
C32	0.0321 (9)	0.0346 (15)	0.0293 (14)	0.0151 (10)	0.0048 (13)	0.0194 (14)
C33	0.0230 (11)	0.0232 (12)	0.0328 (15)	0.0037 (8)	0.0098 (9)	0.0093 (10)
C34	0.0200 (9)	0.0196 (10)	0.0199 (9)	0.0025 (7)	0.0053 (7)	0.0087 (8)
C35	0.0623 (19)	0.0386 (14)	0.0274 (14)	0.0118 (12)	0.0078 (11)	0.0149 (11)
C36	0.0515 (16)	0.0380 (14)	0.0493 (17)	0.0188 (12)	0.0218 (12)	0.0081 (12)
C29B	0.0224 (7)	0.0351 (9)	0.0230 (8)	0.0132 (7)	0.0067 (6)	0.0154 (7)
N3B	0.0277 (11)	0.0317 (13)	0.0181 (9)	0.0151 (8)	0.0019 (8)	0.0086 (9)
C30B	0.0127 (10)	0.0196 (12)	0.0242 (10)	−0.0010 (7)	0.0029 (8)	0.0116 (9)
N4B	0.0223 (7)	0.0286 (18)	0.0277 (14)	0.0028 (12)	0.0026 (10)	0.0109 (10)
C31B	0.0271 (10)	0.0277 (15)	0.0251 (15)	0.0065 (9)	0.0085 (11)	0.0141 (12)
C32B	0.0321 (9)	0.0346 (15)	0.0293 (14)	0.0151 (10)	0.0048 (13)	0.0194 (14)
C33B	0.0230 (11)	0.0232 (12)	0.0328 (15)	0.0037 (8)	0.0098 (9)	0.0093 (10)
C34B	0.0200 (9)	0.0196 (10)	0.0199 (9)	0.0025 (7)	0.0053 (7)	0.0087 (8)
C35B	0.0623 (19)	0.0386 (14)	0.0274 (14)	0.0118 (12)	0.0078 (11)	0.0149 (11)
C36B	0.0515 (16)	0.0380 (14)	0.0493 (17)	0.0188 (12)	0.0218 (12)	0.0081 (12)
C37	0.0252 (7)	0.0257 (8)	0.0213 (7)	0.0020 (6)	0.0051 (6)	0.0120 (6)
C38	0.0317 (8)	0.0410 (10)	0.0212 (8)	−0.0033 (7)	0.0092 (6)	0.0119 (7)
O1A	0.0259 (8)	0.0184 (8)	0.0186 (8)	0.0062 (6)	0.0029 (6)	0.0046 (6)
C1A	0.0240 (9)	0.0193 (10)	0.0187 (11)	0.0009 (7)	−0.0008 (8)	0.0023 (8)
C2A	0.0301 (10)	0.0274 (11)	0.0237 (10)	−0.0051 (8)	0.0030 (8)	0.0049 (8)
C3A	0.0367 (11)	0.0273 (11)	0.0235 (10)	−0.0009 (9)	0.0053 (8)	0.0051 (9)
O1W	0.032 (3)	0.024 (3)	0.028 (4)	0.006 (2)	−0.005 (3)	0.000 (3)

### 1-{[2,6-Bis(hydroxymethyl)-4-methylphenoxy]methyl}-3,5-bis{[(4,6-dimethylpyridin-2-yl)amino]methyl}-2,4,6-triethylbenzene–n-propanol–water (1/0.777/0.223) (1b) Geometric parameters (Å, º)

**Table:**

O1—C8	1.3907 (16)	C27—C28	1.526 (3)
O1—C7	1.4460 (17)	C27—H27A	0.9900
O2—C14	1.4241 (17)	C27—H27B	0.9900
O2—H2	0.850 (9)	C28—H28A	0.9800
O3—C16	1.4179 (17)	C28—H28B	0.9800
O3—H3	0.850 (10)	C28—H28C	0.9800
N1—C20	1.3642 (18)	C29—N3	1.425 (2)
N1—C19	1.4513 (18)	C29—H29A	0.988 (9)
N1—H1B	0.881 (9)	C29—H29B	0.996 (10)
N2—C20	1.3429 (18)	N3—C30	1.372 (3)
N2—C21	1.3599 (18)	N3—H3A	0.883 (9)
C1—C2	1.404 (2)	N4—C30	1.355 (3)
C1—C6	1.4055 (19)	N4—C31	1.362 (3)
C1—C7	1.5154 (18)	C30—C34	1.383 (3)
C2—C3	1.4054 (19)	C31—C32	1.381 (3)
C2—C17	1.5198 (18)	C31—C35	1.493 (3)
C3—C4	1.4043 (19)	C32—C33	1.393 (3)
C3—C19	1.5147 (19)	C32—H32	0.9500
C4—C5	1.399 (2)	C33—C34	1.372 (3)
C4—C27	1.5237 (19)	C33—C36	1.504 (3)
C5—C6	1.4024 (19)	C34—H34	0.9500
C5—C29B	1.5184 (19)	C35—H35A	0.9800
C5—C29	1.5184 (19)	C35—H35B	0.9800
C6—C37	1.516 (2)	C35—H35C	0.9800
C7—H7A	0.9900	C36—H36A	0.9800
C7—H7B	0.9900	C36—H36B	0.9800
C8—C9	1.3958 (18)	C36—H36C	0.9800
C8—C13	1.3975 (18)	C29B—N3B	1.640 (8)
C9—C10	1.3860 (19)	C29B—H29C	0.991 (10)
C9—C14	1.5105 (19)	C29B—H29D	0.994 (11)
C10—C11	1.3942 (19)	N3B—C30B	1.375 (11)
C10—H10	0.9500	N3B—H3B	0.8800
C11—C12	1.3959 (19)	C30B—N4B	1.370 (9)
C11—C15	1.506 (2)	C30B—C34B	1.371 (7)
C12—C13	1.385 (2)	N4B—C31B	1.384 (9)
C12—H12	0.9500	C31B—C32B	1.385 (8)
C13—C16	1.5194 (19)	C31B—C35B	1.518 (8)
C14—H14A	0.9900	C32B—C33B	1.368 (8)
C14—H14B	0.9900	C32B—H32B	0.9500
C15—H15A	0.9800	C33B—C34B	1.371 (7)
C15—H15B	0.9800	C33B—C36B	1.527 (8)
C15—H15C	0.9800	C34B—H34B	0.9500
C16—H16A	0.9900	C35B—H35D	0.9800
C16—H16B	0.9900	C35B—H35E	0.9800
C17—C18	1.535 (2)	C35B—H35F	0.9800
C17—H17A	0.9900	C36B—H36D	0.9800
C17—H17B	0.9900	C36B—H36E	0.9800
C18—H18A	0.9800	C36B—H36F	0.9800
C18—H18B	0.9800	C37—C38	1.528 (2)
C18—H18C	0.9800	C37—H37A	0.9900
C19—H19A	0.9900	C37—H37B	0.9900
C19—H19B	0.9900	C38—H38A	0.9800
C20—C24	1.4073 (19)	C38—H38B	0.9800
C21—C22	1.375 (2)	C38—H38C	0.9800
C21—C25	1.499 (2)	O1A—C1A	1.431 (2)
C22—C23	1.400 (2)	O1A—H1A	0.845 (10)
C22—H22	0.9500	C1A—C2A	1.514 (3)
C23—C24	1.370 (2)	C1A—H1A1	0.9900
C23—C26	1.5030 (19)	C1A—H1A2	0.9900
C24—H24	0.9500	C2A—C3A	1.521 (3)
C25—H25A	0.9800	C2A—H2A1	0.9900
C25—H25B	0.9800	C2A—H2A2	0.9900
C25—H25C	0.9800	C3A—H3A1	0.9800
C26—H26A	0.9800	C3A—H3A2	0.9800
C26—H26B	0.9800	C3A—H3A3	0.9800
C26—H26C	0.9800		
			
C8—O1—C7	115.57 (10)	C4—C27—H27A	109.1
C14—O2—H2	104.6 (15)	C28—C27—H27A	109.1
C16—O3—H3	106.9 (15)	C4—C27—H27B	109.1
C20—N1—C19	122.83 (12)	C28—C27—H27B	109.1
C20—N1—H1B	117.4 (12)	H27A—C27—H27B	107.8
C19—N1—H1B	119.1 (12)	C27—C28—H28A	109.5
C20—N2—C21	117.16 (12)	C27—C28—H28B	109.5
C2—C1—C6	120.70 (12)	H28A—C28—H28B	109.5
C2—C1—C7	121.07 (12)	C27—C28—H28C	109.5
C6—C1—C7	118.23 (12)	H28A—C28—H28C	109.5
C1—C2—C3	119.07 (12)	H28B—C28—H28C	109.5
C1—C2—C17	121.05 (12)	N3—C29—H29A	114.9 (14)
C3—C2—C17	119.86 (12)	N3—C29—H29B	109 (4)
C4—C3—C2	120.64 (12)	H29A—C29—H29B	99 (4)
C4—C3—C19	118.89 (12)	C30—N3—C29	124.39 (17)
C2—C3—C19	120.45 (12)	C30—N3—H3A	116.1 (14)
C5—C4—C3	119.56 (13)	C29—N3—H3A	119.1 (14)
C5—C4—C27	119.92 (12)	C30—N4—C31	116.3 (2)
C3—C4—C27	120.50 (13)	N4—C30—N3	117.7 (2)
C4—C5—C6	120.59 (12)	N4—C30—C34	124.2 (2)
C5—C6—C1	119.36 (13)	N3—C30—C34	118.01 (18)
C5—C6—C37	120.51 (12)	N4—C31—C32	122.3 (2)
C1—C6—C37	120.13 (12)	N4—C31—C35	116.1 (2)
O1—C7—C1	113.97 (11)	C32—C31—C35	121.5 (2)
O1—C7—H7A	108.8	C31—C32—C33	119.9 (2)
C1—C7—H7A	108.8	C31—C32—H32	120.0
O1—C7—H7B	108.8	C33—C32—H32	120.0
C1—C7—H7B	108.8	C34—C33—C32	118.57 (19)
H7A—C7—H7B	107.7	C34—C33—C36	119.4 (2)
O1—C8—C9	118.59 (11)	C32—C33—C36	122.0 (2)
O1—C8—C13	119.47 (11)	C33—C34—C30	118.60 (18)
C9—C8—C13	121.57 (12)	C33—C34—H34	120.7
C10—C9—C8	118.17 (12)	C30—C34—H34	120.7
C10—C9—C14	122.45 (12)	C31—C35—H35A	109.5
C8—C9—C14	119.34 (12)	C31—C35—H35B	109.5
C9—C10—C11	121.80 (13)	H35A—C35—H35B	109.5
C9—C10—H10	119.1	C31—C35—H35C	109.5
C11—C10—H10	119.1	H35A—C35—H35C	109.5
C10—C11—C12	118.43 (13)	H35B—C35—H35C	109.5
C10—C11—C15	120.63 (13)	C33—C36—H36A	109.5
C12—C11—C15	120.93 (12)	C33—C36—H36B	109.5
C13—C12—C11	121.44 (12)	H36A—C36—H36B	109.5
C13—C12—H12	119.3	C33—C36—H36C	109.5
C11—C12—H12	119.3	H36A—C36—H36C	109.5
C12—C13—C8	118.48 (12)	H36B—C36—H36C	109.5
C12—C13—C16	122.44 (12)	N3B—C29B—H29C	79 (6)
C8—C13—C16	119.01 (12)	N3B—C29B—H29D	128 (7)
O2—C14—C9	111.93 (12)	H29C—C29B—H29D	133 (9)
O2—C14—H14A	109.2	C30B—N3B—C29B	117.4 (6)
C9—C14—H14A	109.2	C30B—N3B—H3B	121.3
O2—C14—H14B	109.2	C29B—N3B—H3B	121.3
C9—C14—H14B	109.2	N4B—C30B—C34B	125.1 (8)
H14A—C14—H14B	107.9	N4B—C30B—N3B	122.1 (8)
C11—C15—H15A	109.5	C34B—C30B—N3B	112.8 (6)
C11—C15—H15B	109.5	C30B—N4B—C31B	117.6 (9)
H15A—C15—H15B	109.5	N4B—C31B—C32B	118.1 (9)
C11—C15—H15C	109.5	N4B—C31B—C35B	120.5 (8)
H15A—C15—H15C	109.5	C32B—C31B—C35B	121.4 (9)
H15B—C15—H15C	109.5	C33B—C32B—C31B	122.3 (9)
O3—C16—C13	113.73 (12)	C33B—C32B—H32B	118.9
O3—C16—H16A	108.8	C31B—C32B—H32B	118.9
C13—C16—H16A	108.8	C32B—C33B—C34B	120.4 (7)
O3—C16—H16B	108.8	C32B—C33B—C36B	126.8 (8)
C13—C16—H16B	108.8	C34B—C33B—C36B	112.7 (7)
H16A—C16—H16B	107.7	C30B—C34B—C33B	116.4 (6)
C2—C17—C18	112.02 (12)	C30B—C34B—H34B	121.8
C2—C17—H17A	109.2	C33B—C34B—H34B	121.8
C18—C17—H17A	109.2	C31B—C35B—H35D	109.5
C2—C17—H17B	109.2	C31B—C35B—H35E	109.5
C18—C17—H17B	109.2	H35D—C35B—H35E	109.5
H17A—C17—H17B	107.9	C31B—C35B—H35F	109.5
C17—C18—H18A	109.5	H35D—C35B—H35F	109.5
C17—C18—H18B	109.5	H35E—C35B—H35F	109.5
H18A—C18—H18B	109.5	C33B—C36B—H36D	109.5
C17—C18—H18C	109.5	C33B—C36B—H36E	109.5
H18A—C18—H18C	109.5	H36D—C36B—H36E	109.5
H18B—C18—H18C	109.5	C33B—C36B—H36F	109.5
N1—C19—C3	109.77 (12)	H36D—C36B—H36F	109.5
N1—C19—H19A	109.7	H36E—C36B—H36F	109.5
C3—C19—H19A	109.7	C6—C37—C38	112.42 (13)
N1—C19—H19B	109.7	C6—C37—H37A	109.1
C3—C19—H19B	109.7	C38—C37—H37A	109.1
H19A—C19—H19B	108.2	C6—C37—H37B	109.1
N2—C20—N1	117.99 (12)	C38—C37—H37B	109.1
N2—C20—C24	122.90 (12)	H37A—C37—H37B	107.9
N1—C20—C24	119.11 (13)	C37—C38—H38A	109.5
N2—C21—C22	122.59 (13)	C37—C38—H38B	109.5
N2—C21—C25	116.06 (13)	H38A—C38—H38B	109.5
C22—C21—C25	121.34 (13)	C37—C38—H38C	109.5
C21—C22—C23	120.18 (13)	H38A—C38—H38C	109.5
C21—C22—H22	119.9	H38B—C38—H38C	109.5
C23—C22—H22	119.9	C1A—O1A—H1A	106.5 (18)
C24—C23—C22	117.67 (13)	O1A—C1A—C2A	111.33 (15)
C24—C23—C26	121.49 (13)	O1A—C1A—H1A1	109.4
C22—C23—C26	120.81 (13)	C2A—C1A—H1A1	109.4
C23—C24—C20	119.50 (13)	O1A—C1A—H1A2	109.4
C23—C24—H24	120.3	C2A—C1A—H1A2	109.4
C20—C24—H24	120.3	H1A1—C1A—H1A2	108.0
C21—C25—H25A	109.5	C1A—C2A—C3A	112.71 (17)
C21—C25—H25B	109.5	C1A—C2A—H2A1	109.0
H25A—C25—H25B	109.5	C3A—C2A—H2A1	109.0
C21—C25—H25C	109.5	C1A—C2A—H2A2	109.0
H25A—C25—H25C	109.5	C3A—C2A—H2A2	109.0
H25B—C25—H25C	109.5	H2A1—C2A—H2A2	107.8
C23—C26—H26A	109.5	C2A—C3A—H3A1	109.5
C23—C26—H26B	109.5	C2A—C3A—H3A2	109.5
H26A—C26—H26B	109.5	H3A1—C3A—H3A2	109.5
C23—C26—H26C	109.5	C2A—C3A—H3A3	109.5
H26A—C26—H26C	109.5	H3A1—C3A—H3A3	109.5
H26B—C26—H26C	109.5	H3A2—C3A—H3A3	109.5
C4—C27—C28	112.48 (13)		
			
C6—C1—C2—C3	3.2 (2)	C2—C3—C19—N1	94.74 (15)
C7—C1—C2—C3	−176.64 (12)	C21—N2—C20—N1	−179.71 (12)
C6—C1—C2—C17	−178.39 (13)	C21—N2—C20—C24	−0.16 (19)
C7—C1—C2—C17	1.8 (2)	C19—N1—C20—N2	8.0 (2)
C1—C2—C3—C4	−1.6 (2)	C19—N1—C20—C24	−171.53 (13)
C17—C2—C3—C4	179.94 (13)	C20—N2—C21—C22	0.3 (2)
C1—C2—C3—C19	−179.86 (12)	C20—N2—C21—C25	−179.39 (13)
C17—C2—C3—C19	1.7 (2)	N2—C21—C22—C23	0.0 (2)
C2—C3—C4—C5	0.0 (2)	C25—C21—C22—C23	179.73 (14)
C19—C3—C4—C5	178.30 (13)	C21—C22—C23—C24	−0.5 (2)
C2—C3—C4—C27	178.44 (13)	C21—C22—C23—C26	177.74 (13)
C19—C3—C4—C27	−3.3 (2)	C22—C23—C24—C20	0.69 (19)
C3—C4—C5—C6	0.0 (2)	C26—C23—C24—C20	−177.58 (13)
C27—C4—C5—C6	−178.39 (13)	N2—C20—C24—C23	−0.4 (2)
C4—C5—C6—C1	1.5 (2)	N1—C20—C24—C23	179.19 (12)
C4—C5—C6—C37	−178.81 (13)	C5—C4—C27—C28	88.08 (17)
C2—C1—C6—C5	−3.1 (2)	C3—C4—C27—C28	−90.33 (17)
C7—C1—C6—C5	176.69 (13)	C31—N4—C30—N3	−177.1 (3)
C2—C1—C6—C37	177.18 (13)	C31—N4—C30—C34	1.1 (5)
C7—C1—C6—C37	−3.00 (19)	C29—N3—C30—N4	3.9 (3)
C8—O1—C7—C1	81.11 (14)	C29—N3—C30—C34	−174.49 (19)
C2—C1—C7—O1	−106.00 (15)	C30—N4—C31—C32	1.3 (5)
C6—C1—C7—O1	74.17 (16)	C30—N4—C31—C35	−176.5 (3)
C7—O1—C8—C9	−106.69 (14)	N4—C31—C32—C33	−2.6 (5)
C7—O1—C8—C13	80.09 (15)	C35—C31—C32—C33	175.1 (3)
O1—C8—C9—C10	−175.59 (12)	C31—C32—C33—C34	1.5 (4)
C13—C8—C9—C10	−2.5 (2)	C31—C32—C33—C36	179.0 (3)
O1—C8—C9—C14	2.16 (19)	C32—C33—C34—C30	0.7 (3)
C13—C8—C9—C14	175.23 (13)	C36—C33—C34—C30	−176.9 (2)
C8—C9—C10—C11	−0.5 (2)	N4—C30—C34—C33	−2.1 (4)
C14—C9—C10—C11	−178.21 (14)	N3—C30—C34—C33	176.15 (19)
C9—C10—C11—C12	2.4 (2)	C29B—N3B—C30B—N4B	−12.4 (13)
C9—C10—C11—C15	−178.90 (14)	C29B—N3B—C30B—C34B	167.6 (6)
C10—C11—C12—C13	−1.2 (2)	C34B—C30B—N4B—C31B	−2 (2)
C15—C11—C12—C13	−179.96 (13)	N3B—C30B—N4B—C31B	177.6 (11)
C11—C12—C13—C8	−1.7 (2)	C30B—N4B—C31B—C32B	−1 (2)
C11—C12—C13—C16	175.12 (13)	C30B—N4B—C31B—C35B	178.6 (12)
O1—C8—C13—C12	176.62 (12)	N4B—C31B—C32B—C33B	3 (2)
C9—C8—C13—C12	3.6 (2)	C35B—C31B—C32B—C33B	−176.2 (11)
O1—C8—C13—C16	−0.29 (19)	C31B—C32B—C33B—C34B	−2.4 (17)
C9—C8—C13—C16	−173.30 (13)	C31B—C32B—C33B—C36B	−178.8 (12)
C10—C9—C14—O2	−5.2 (2)	N4B—C30B—C34B—C33B	3.4 (14)
C8—C9—C14—O2	177.20 (13)	N3B—C30B—C34B—C33B	−176.6 (7)
C12—C13—C16—O3	−2.0 (2)	C32B—C33B—C34B—C30B	−0.9 (12)
C8—C13—C16—O3	174.78 (12)	C36B—C33B—C34B—C30B	175.9 (7)
C1—C2—C17—C18	−89.61 (16)	C5—C6—C37—C38	−86.85 (16)
C3—C2—C17—C18	88.81 (16)	C1—C6—C37—C38	92.84 (16)
C20—N1—C19—C3	163.00 (12)	O1A—C1A—C2A—C3A	178.58 (17)
C4—C3—C19—N1	−83.54 (16)		

### 1-{[2,6-Bis(hydroxymethyl)-4-methylphenoxy]methyl}-3,5-bis{[(4,6-dimethylpyridin-2-yl)amino]methyl}-2,4,6-triethylbenzene–n-propanol–water (1/0.777/0.223) (1b) Hydrogen-bond geometry (Å, º)

*Cg*1, *Cg*2 and *Cg*3 represent the centroids of the C1–C6, C8–C13 and C20–C24/N2 rings, respectively.

**Table:**

*D*—H···*A*	*D*—H	H···*A*	*D*···*A*	*D*—H···*A*
N1—H1*B*···O3^i^	0.88 (1)	2.21 (1)	3.0704 (18)	165 (2)
O2—H2···O1*A*	0.85 (1)	1.86 (2)	2.702 (2)	171 (2)
O2—H2···O1*W*	0.85 (1)	1.97 (2)	2.773 (6)	158 (2)
O3—H3···O2^ii^	0.85 (1)	1.92 (2)	2.7646 (15)	171 (2)
O1*A*—H1*A*···N2^iii^	0.85 (1)	1.97 (2)	2.813 (2)	177 (2)
C10—H10···O2	0.95	2.44	2.7925 (17)	102
C12—H12···O3	0.95	2.49	2.8362 (17)	101
C24—H24···O3^i^	0.95	2.65	3.4259 (19)	139
C27—H27*A*···N3	0.99	2.55	3.257 (3)	128
C34*B*—H34*B*···O1*W*	0.95	2.16	2.969 (9)	143
C37—H37*A*···O1	0.99	2.41	3.1687 (17)	133
C14—H14*B*···*Cg*1	0.99	2.88	3.8427 (18)	166
C25—H25*B*···*Cg*2^iv^	0.98	2.72	3.4520 (18)	132
C18—H18*B*···*Cg*3^ii^	0.98	2.69	3.6317 (16)	161

Symmetry codes: (i) *x*−1, *y*, *z*; (ii) *x*+1, *y*, *z*; (iii) *x*, *y*+1, *z*; (iv) *x*−1, *y*−1, *z*.
